# Associations of non‐alcoholic fatty liver disease and cirrhosis with liver cancer in European and East Asian populations: A Mendelian randomization study

**DOI:** 10.1002/cnr2.1913

**Published:** 2023-10-16

**Authors:** Yunyang Deng, Junjie Huang, Martin Chi Sang Wong

**Affiliations:** ^1^ The Jockey Club School of Public Health and Primary Care, Faculty of Medicine The Chinese University of Hong Kong Hong Kong SAR China; ^2^ School of Public Health The Chinese Academy of Medical Sciences and Peking Union Medical College Beijing China; ^3^ School of Public Health Peking University Beijing China

**Keywords:** cirrhosis, hepatocellular carcinoma, liver cancer, Mendelian randomization, non‐alcoholic fatty liver disease

## Abstract

**Background:**

The positive relationships of non‐alcoholic fatty liver disease (NAFLD) and cirrhosis with liver cancer were shown in previous observational studies, while further Mendelian randomization (MR) investigations are needed to confirm the possible causal associations.

**Aims:**

This study aimed to explore whether NAFLD and cirrhosis were causally related to liver cancer using MR in European and East Asian populations.

**Methods and Results:**

For European populations, NAFLD data were obtained from a genome‐wide meta‐analysis (8434 patients and 770 180 controls). The data on chronic elevation of alanine aminotransferase (cALT), a proxy of NAFLD, were derived from Million Veteran Program (68 725 patients and 95 472 controls). Cirrhosis data were collected from two sources: a genome‐wide association study of five cohorts (4829 patients and 72 705 controls) and FinnGen (1931 patients and 216 861 controls). Liver cancer data were collected from FinnGen (304 patients and 174 006 controls). For East Asian populations, the data on cirrhosis (2184 patients and 210 269 controls) and hepatocellular carcinoma (1866 patients and 195 745 controls) were obtained from Biobank Japan.

Three, 41, seven, six, and three single‐nucleotide polymorphisms were used for NAFLD (European), cALT (European), cirrhosis (European‐five cohorts), cirrhosis (European‐FinnGen), and cirrhosis (East Asian), respectively. We used inverse‐variance weighted as the primary method to calculate the odds ratio (OR) and 95% confidence interval (CI).

Among European populations, genetically‐predicted NAFLD, cALT, cirrhosis (five cohorts), and cirrhosis (FinnGen) were positively associated with liver cancer, with ORs (95% CIs) of 6.62 (3.81–11.50) (*p* < .001), 2.59 (1.70–3.94) (*p* < .001), 3.38 (2.41–4.75) (*p* < .001), and 2.62 (1.20–5.72) (*p* = .015). Among East Asian populations, there was also a positive association between genetically‐predicted cirrhosis and hepatocellular carcinoma (OR = 2.12; 95% CI = 1.78–2.52; *p* < .001).

**Conclusion:**

This study utilized MR to complementarily confirm the positive connections of NAFLD and cirrhosis with liver cancer, as identified in earlier observational research. Subsequent MR investigations involving more liver cancer cases are needed.

## 1 INTRODUCTION

Globally, liver cancer ranked sixth for cancer incidence and third for cancer death in 2020.^1^ Hepatocellular carcinoma (HCC) was the dominant type for liver cancer.^1^ Liver cancer risk factors included hepatitis virus, aflatoxin B1, alcohol, unhealthy diet, diabetes, obesity, non‐alcoholic fatty liver disease (NAFLD), and cirrhosis.^2^, ^3^ NAFLD was defined by the presence of steatosis in over 5% of hepatocytes based on histology without excessive alcohol intake (men: ≥30 g/day; women: ≥20 g/day) and other chronic liver diseases.^4^ It was the most common chronic hepatic disorder with a prevalence of 25%,^5^ which was likely driven by genetic composition, high caloric intake, physical inactivity, and some socioeconomic factors.^6^, ^7^ Cirrhosis was characterized by diffuse hepatic fibrosis and the replacement of normal liver structures with regenerating liver nodules.^8^ Although cirrhosis mortality declined from 1990 to 2017, the numbers of deaths and the proportion of deaths due to cirrhosis increased during the same period.^9^

Prior observational investigations have shown that NAFLD and cirrhosis were positively associated with liver cancer. A systematic review and meta‐analysis of 103 observational studies found that NAFLD (vs. non‐NAFLD) can increase 88% HCC risk.^10^ Another systematic review and meta‐analysis of 18 studies showed that people with cirrhosis had a higher HCC incidence than those without (3.78 vs. 0.03 per 100 person‐years).^11^ A Japanese review of 34 studies revealed that HCC ranked the first for cancer incidence and mortality in NAFLD patients.^12^

However, the intrinsic limitations of observational studies (uncontrolled confounders and reverse causality) prevent them from being used to establish causal relationships.^13^, ^14^, ^15^ Mendelian randomization (MR) is a tool for inferring causality, which exploits genetic variants like single‐nucleotide polymorphisms (SNPs) as instrumental variables (IVs) to investigate the potential causal relationships.^13^, ^14^, ^15^ Since genetic variants are randomly assigned during meiosis, MR may partially overcome the drawbacks of observational studies.^13^, ^14^, ^15^ Nonetheless, to our knowledge, no MR investigations have been conducted to explore whether NAFLD and cirrhosis were causally related to liver cancer. Thus, it is warrant to perform relevant MR research to complementarily support the correlations found in previous observational studies.

The purpose of this study was to assess the possible causal relationships of NAFLD and cirrhosis with liver cancer in European and East Asian populations by MR analyses.

## 2 METHODS

This MR study adhered to the guideline of strengthening the reporting of observational studies in epidemiology using Mendelian randomization (STROBE‐MR) (Table S1).^14^ All participants in the previous genome‐wide association studies (GWASs) provided their written informed consents.^16^, ^17^, ^18^, ^19^, ^20^, ^21^ The respective ethics review boards had granted approval for all GWASs.^16^, ^17^, ^18^, ^19^, ^20^, ^21^ This study remained within the boundaries set by the initial approvals from the ethics committees.

### 2.1 Data sources for European populations

Public summarized GWAS data on NAFLD were derived from a genome‐wide meta‐analysis with 778 614 participants (8434 patients and 770 180 controls). The meta‐analysis was based on four European consortiums, including electronic Medical Records and Genomics (eMERGE) Network (1106 patients and 8571 controls), United Kingdom Biobank (UKB) (2558 patients and 395 241 controls), Estonian Biobank (4119 patients and 190 120 controls), and FinnGen (651 patients and 176 248 controls) (Table 1).^16^

**TABLE 1:** Details of data sources in this study.

Traits	Consortium	Year	Sample size (cases/controls)	GWAS ID^a^	PubMed ID
European populations					
Non‐alcoholic fatty liver disease	A meta‐analysis	2021	778 614 (8434/770180)	/	34841290
cALT	MVP	2023	164 197 (68 725/95472)	/	35654975
Cirrhosis (five cohorts)	A GWAS of five cohorts	2021	77 534 (4829/72705)	/	33310085
Cirrhosis (FinnGen)	FinnGen	2021	218 792 (1931/216861)	finn‐b‐CIRRHOSIS_BROAD	/
Liver and intrahepatic bile ducts cancer	FinnGen	2021	174 310 (304/174006)	finn‐b‐C3_LIVER_INTRAHEPATIC_BILE_DUCTS_EXALLC	/
Overweight	GIANT	2013	158 855 (93 015/65840)	ieu‐a‐93	23563607
Obesity class 1	GIANT	2013	98 697 (32 858/65839)	ieu‐a‐90	23563607
Obesity class 2	GIANT	2013	72 546 (9889/62657)	ieu‐a‐91	23563607
Obesity class 3	GIANT	2013	50 364 (2896/47468)	ieu‐a‐92	23563607
Ever versus never smokers	TAG	2010	74 035 (41 969/32066)	ieu‐a‐962	20418890
Cigarettes consumption (per day)	GSCAN	2019	249 752	ieu‐b‐142	30643251
Alcohol consumption (per week)	GSCAN	2019	335 394	ieu‐b‐73	30643251
Hypertension	UKB	2018	361 194 (1237/359957)	ukb‐d‐I9_HYPTENS	/
Coronary artery disease	CARDIoGRAMplusC4D	2015	184 305 (60 801/123504)	ieu‐a‐7	26343387
Type 2 diabetes	DIAGRAM	2014	110 452 (26 488/83964)	ieu‐a‐23	24509480
East Asian populations					
Cirrhosis	Biobank Japan	2020	212 453 (2184/210269)	bbj‐a‐105	32514122
Hepatocellular carcinoma	Biobank Japan	2020	197 611 (1866/195745)	bbj‐a‐158	32514122
Body mass index	Biobank Japan	2019	158 284	bbj‐a‐1	28892062
Smoking	Biobank Japan	2020	165 436	bbj‐a‐78	31089300
Total cholesterol	Biobank Japan	2020	128 305	bbj‐a‐54	29403010
Low‐density lipoprotein cholesterol	Biobank Japan	2020	72 866	bbj‐a‐31	29403010
High‐density lipoprotein cholesterol	Biobank Japan	2020	70 657	bbj‐a‐24	29403010
Diastolic blood pressure	Biobank Japan	2020	136 615	bbj‐a‐17	29403010
Systolic blood pressure	Biobank Japan	2020	136 597	bbj‐a‐52	29403010
Mean arterial pressure	Biobank Japan	2020	136 482	bbj‐a‐37	29403010
Congestive heart failure	Biobank Japan	2020	297 453 (94 413/203040)	bbj‐a‐109	32514122
Type 2 diabetes	Biobank Japan	2020	210 865 (40 250/170615)	bbj‐a‐153	32514122
Chronic hepatitis B	Biobank Japan	2020	212 453	bbj‐a‐99	32514122
Chronic hepatitis C	Biobank Japan	2020	212 453	bbj‐a‐101	32514122
Ever/never alcohol drinker	UKB (EAS)	2020	2656 (1983/673)	ukb‐e‐20117_p3_EAS	/

Abbreviations: cALT, chronic elevation of alanine aminotransferase; CARDIoGRAMplusC4D, Coronary ARtery DIsease Genome wide Replication and Meta‐analysis plus The Coronary Artery Disease; DIAGRAM, DIAbetes Genetics Replication and Meta‐analysis; EAS, East Asian; GIANT, Genetic Investigation of ANthropometric Traits; GSCAN, GWAS and Sequencing Consortium of Alcohol and Nicotine use; GWAS, genome‐wide association study; MVP, Million Veteran Program; TAG, Tobacco and Genetics; UKB, United Kingdom Biobank.

^a^ GWAS ID in the MR‐Base database.

The eMERGE Network is a large‐scale genetic research institution consists of 10 US sites.^17^ The ethic review boards at each site where participants were recruited approved the GWAS and all subjects provided written informed consents.^17^ The eMERGE Network included 9677 European participants (1106 patients and 8571 controls) for NAFLD GWAS.^17^ NAFLD was diagnosed based on International Classification of Diseases (ICD) codes, including ICD9 (571.5, 571.8, and 571.9) and ICD10 (K75.81, K76.0, and K76.9).^17^ Participants were excluded if they had some liver‐related diseases (e.g., hepatitis, biliary atresia, and alcoholic fatty liver) and related medications (e.g., atazanavir, ritonavir, and indinavir).^17^ Details of the inclusion and exclusion criteria can be found in a previous study.^17^ GWAS was conducted with covariates being age, sex, the top three principal components (PCs), and 10 sites.^16^, ^17^ Participants with any missing data were excluded from the GWAS.^16^, ^17^ The reference genome build was HG19/GRCh37.^16^, ^17^ Other details of GWAS (e.g., Manhattan plots, genotyping and imputation, etc.) can be found in the original GWASs.^16^, ^17^

UKB is a large biobank including over 500 000 UK individuals.^16^ UKB‐related GWAS were approved by North West Multi‐center Research Ethics Committee and written informed consents were collected from all participants.^16^ In the genome‐wide meta‐analysis, the authors conducted a new GWAS for NAFLD using data from UKB (2558 cases and 395 241 controls).^16^ NAFLD was diagnosed based on medical records (ICD10: K74.0, K74.2, K75.8, K76.0, and K76.9).^16^ The exclusion criteria were the same as those applied in eMERGE Network.^16^ GWAS was performed with age, sex, and the top 10 PCs as the covariates.^16^ Subjects with missing data were excluded from the GWAS.^16^ The reference genome build was HG19/GRCh37.^16^ Other details of GWAS were shown in the original GWAS.^16^

Estonian Biobank is a population‐based biobank with over 200 000 Estonian adults.^16^ All participants signed consents before recruitment.^16^ Similar with UKB, the genome‐wide meta‐analysis performed a new GWAS for NAFLD based on Estonian Biobank, which was approved by Research Ethics Committee of the University of Tartu.^16^ 194 239 European individuals (4119 cases and 190 120 controls) from Estonian Biobank were included, with the same inclusion/exclusion criteria and GWAS procedures as those employed in UKB.^16^ Subjects with any missing data were excluded.^16^ The reference genome build was HG19/GRCh37.^16^ Other GWAS details can be found in the original GWAS.^16^

FinnGen is a public‐private project aims to explore the associations between genetic variations and human health in European participants.^18^ In the genome‐wide meta‐analysis, the authors obtained summarized GWAS data on NAFLD from FinnGen (651 cases and 176 248 controls).^16^ NAFLD was diagnosed based on ICD‐10 (K76.0).^16^ Age, sex, the top 10 PCs, and genotyping batches were included as covariates in the GWAS.^16^, ^18^ The reference genome build was HG19/GRCh37.^16^, ^18^ Other GWAS details were shown in the original GWAS.^16^, ^18^

In addition, we collected public summarized GWAS data on chronic elevation of alanine aminotransferase (cALT), a proxy of NAFLD, from the Million Veteran Program (MVP).^19^ The MVP, which began in 2011, is a biobank funded by the US Veterans Health Administration Office of Research and Development.^19^ The VA Central Institutional Review Board granted ethical approval for the MVP.^19^ In the MVP, cALT level was measured as a dichotomous variable (cALT cases and controls).^19^ The cALT case was characterized as a cALT level higher than 40 U/L for males or over 30 U/L for females on at least two occasions separated by at least 6 months within a span of 2 years before joining the study.^19^ A normal cALT was used to identify the control group (males: 30 U/L; females: 20 U/L).^19^ Individuals with chronic liver diseases and/or alcohol‐related disorders were excluded.^19^ Finally, the MVP included 164 197 European Americans, including 68 725 cALT cases aged 63.1 ± 11.9 years and 95 472 controls aged 67.8 ± 13.5 years.^19^ The covariates were age, gender, age‐adjusted Alcohol Use Disorders Identification Test score, and the top 10 PCs.^19^ The reference genome build was HG19/GRCh37.^19^ Other GWAS details can be found in the original GWAS.^19^

For cirrhosis, the public summarized GWAS data were derived from a GWAS of five European cohorts (4829 cirrhosis cases and 72 705 controls), including UKB (2701 cases and 16 206 controls), Atherosclerosis Risk in Communities study (88 cases and 10 034 controls), Vanderbilt BioVU (1328 cases and 45 000 controls), a German cohort (410 cases and 1119 controls), and an alcoholic cirrhosis GWAS (302 cases and 346 controls).^20^ All cohorts were approved by appropriate ethics review boards.^20^ Cirrhosis was diagnosed using ICD codes of K70.2, K70.3, K70.4, K74.0, K74.1, K74.2, K74.6, K76.6, and I85.^20^ GWASs were performed with covariates of age, sex, and the top 10 PCs.^20^ The reference genome build was HG19/GRCh37.^20^ Other GWAS details were shown in the original GWAS.^20^

Meanwhile, we obtained public summarized GWAS data on cirrhosis (1931 cases and 216 861 controls) and liver and intrahepatic bile ducts cancer (304 cases and 174 006 controls) from FinnGen.^18^ The diagnosed codes of cirrhosis were K70.2, K70.3, K70.4, K74.0, K74.1, K74.2, K74.6, K76.6, and I85.^18^ Liver cancer was diagnosed based on Finnish nationwide medical records and participants with cancers were excluded from controls.^18^ The covariates were age, sex, the top 10 PCs, and genotyping batches.^18^ The reference genome build was HG19/GRCh37.^18^ Other GWAS details can be found in the original GWAS.^18^

### 2.2 Data sources for East Asian populations

Public summarized GWAS data on cirrhosis (2184 patients and 210 269 controls) and HCC (1866 patients and 195 745 controls) were obtained from Biobank Japan (BBJ) (Table 1).^21^ BBJ serves as the largest East Asian biobank which consists of more than 200 000 Japanese individuals aged 20–89 years who were followed up in 12 medical institutions from 2003 to 2018.^21^ Written informed consents were obtained from all participants.^21^ Related GWASs were approved by the ethical committees of RIKEN Yokohama Institute and the Institute of Medical Science, the University of Tokyo.^21^

For cirrhosis, 2184 patients aged 65.7 ± 10.1 years (1347 males and 837 females) and 210 269 controls aged 61.9 ± 13.8 years (108 000 males and 102 269 females) were included.^21^ For HCC, 1866 patients aged 68.0 ± 8.4 years (1384 males and 482 females) and 195 745 controls aged 61.6 ± 13.7 years (97 655 males and 98 090 females) were involved.^21^ Experienced medical professionals at collaborating institutions were responsible for diagnosing the illnesses.^21^ Individuals without any medical conditions were enlisted from BBJ and four additional Japanese cohorts to serve as the control group.^21^ GWASs were performed by adjusting for age, sex, and the top five PCs.^21^ The reference genome build was HG19/GRCh37.^21^ Other GWAS details were shown in the original GWAS.^21^

### 2.3 Instrumental variables selection

SNPs met the following criteria were identified as IVs^13^, ^14^, ^15^: (1) were associated with exposures (*P* < 5 × 10^−8^); (2) were independent (linkage disequilibrium‐*r* ^2^ < 0.001 within 10 000 kilobases); (3) were not palindromic (A/T or C/G) with intermediated effect allele frequencies (40–70%). Notably, we selected SNPs with a more relaxed threshold (*P* < 1 × 10^−6^) as IVs for cirrhosis (European populations‐FinnGen) because limited SNPs met the conventional threshold (5 × 10^−8^). Finally, three, 41, seven, six, and three SNPs were used for NAFLD (European populations), cALT (European populations), cirrhosis (European populations‐five cohorts), cirrhosis (European populations‐FinnGen), and cirrhosis (East Asian populations), respectively. Details of used SNPs can be found in Table 2.

**TABLE 2:** Characteristics of SNPs associated with NAFLD, cALT, and cirrhosis in European and East Asian populations.

SNP	CHR	Position	Gene	EA/NEA	EAF	Beta	SE	*p*	Sample size (cases/controls)	*F*
NAFLD (European populations)										
rs73001065	19	19 460 541	TM6SF2	C/G	0.065	0.281	0.033	7.36E‐18	778 614 (8434/770180)	74
rs429358	19	45 411 941	APOE	T/C	0.932	0.137	0.024	1.14E‐08	778 614 (8434/770180)	33
rs3747207	22	44 324 855	PNPLA3	A/G	0.222	0.289	0.020	5.07E‐48	778 614 (8434/770180)	212
cALT (European populations)										
rs10201587	2	202 202 791	CASP8	G/A	0.499	−0.045	0.007	4.11E‐10	164 197 (68 725/95472)	41
rs10883451	10	101 924 418	ERLIN1	C/T	0.477	−0.161	0.007	3.60E‐106	164 197 (68 725/95472)	529
rs112128680	16	72 054 052	DHODH;HP;HPR	A/G	0.149	0.061	0.010	3.70E‐09	164 197 (68 725/95472)	37
rs11601507	11	5 701 074	TRIM5	A/C	0.073	0.088	0.014	2.16E‐10	164 197 (68 725/95472)	40
rs11621792	14	24 871 926	NFATC4	T/C	0.453	0.042	0.007	1.11E‐08	164 197 (68 725/95472)	36
rs1169292	12	121 426 478	P2RX7;HNF1A	T/C	0.321	0.054	0.008	4.45E‐12	164 197 (68 725/95472)	46
rs12500824	4	77 416 627	SHROOM3	A/G	0.350	0.046	0.008	1.37E‐09	164 197 (68 725/95472)	33
rs132665	22	36 564 170	APOL3	G/A	0.154	−0.069	0.010	1.84E‐11	164 197 (68 725/95472)	48
rs13409360	2	113 838 102	IL1RN	A/G	0.397	−0.060	0.008	1.95E‐15	164 197 (68 725/95472)	56
rs1497406	1	16 505 320	EPHA2	A/G	0.426	−0.042	0.007	1.46E‐08	164 197 (68 725/95472)	36
rs1547014	22	29 100 711	CHEK2	T/C	0.302	−0.064	0.008	5.31E‐16	164 197 (68 725/95472)	64
rs1594320	3	149 114 757	TM4SF1;CP;WWTR1	G/C	0.296	0.053	0.008	5.25E‐11	164 197 (68 725/95472)	44
rs168144	15	60 914 262	RORA;ANXA2	C/T	0.433	−0.052	0.007	3.45E‐12	164 197 (68 725/95472)	55
rs174535	11	61 551 356	FADS1;FADS2;FADS3	C/T	0.337	−0.061	0.008	3.11E‐15	164 197 (68 725/95472)	58
rs17780834	10	104 091 096	FBXL15;ELOVL3;PSD	T/A	0.061	0.086	0.015	1.65E‐08	164 197 (68 725/95472)	33
rs1801689	17	64 210 580	APOH	C/A	0.032	0.176	0.021	1.39E‐17	164 197 (68 725/95472)	70
rs2138157	2	227 103 717	IRS1;MIR5702	A/C	0.353	−0.064	0.008	3.49E‐17	164 197 (68 725/95472)	64
rs2377957	20	32 554 473	AHCY;ITCH	A/G	0.329	−0.053	0.008	1.53E‐11	164 197 (68 725/95472)	44
rs2642438	1	220 970 028	MTARC1	A/G	0.295	−0.079	0.008	8.04E‐23	164 197 (68 725/95472)	98
rs2792751	10	113 940 329	GPAM	T/C	0.293	0.072	0.008	1.28E‐19	164 197 (68 725/95472)	81
rs28929474	14	94 844 947	SERPINA1;SERPINA6	T/C	0.018	0.481	0.028	1.03E‐65	164 197 (68 725/95472)	295
rs2954038	8	126 507 389	TRIB1	C/A	0.306	0.139	0.008	2.12E‐70	164 197 (68 725/95472)	302
rs3810367	19	4 342 847	SIRT6;STAP2	G/T	0.376	0.045	0.008	3.51E‐09	164 197 (68 725/95472)	32
rs4148824	7	87 075 362	ABCB4	G/A	0.185	−0.057	0.010	2.62E‐09	164 197 (68 725/95472)	32
rs429358	19	45 411 941	APOE;APOC1	C/T	0.139	−0.094	0.011	4.72E‐19	164 197 (68 725/95472)	73
rs4684847	3	12 386 337	PPARG	T/C	0.123	−0.072	0.011	1.11E‐10	164 197 (68 725/95472)	43
rs4734654	8	103 669 991	KLF10	G/A	0.362	−0.052	0.008	1.48E‐11	164 197 (68 725/95472)	42
rs4841133	8	9 183 664	PPP1R3B;TNKS;MFHAS1	A/G	0.088	0.130	0.013	1.02E‐24	164 197 (68 725/95472)	100
rs4919741	12	53 272 920	KRT84;KRT74	A/G	0.344	−0.057	0.008	1.72E‐13	164 197 (68 725/95472)	51
rs4940689	18	56 089 116	‐	A/G	0.205	0.054	0.010	1.21E‐08	164 197 (68 725/95472)	29
rs55868793	15	73 956 856	CD276	G/T	0.408	0.059	0.008	8.92E‐15	164 197 (68 725/95472)	54
rs574044675	3	172 274 232	TNFSF10	C/A	0.024	−0.251	0.027	5.07E‐20	164 197 (68 725/95472)	86
rs58542926	19	19 379 549	TM6SF2	T/C	0.075	0.222	0.014	2.13E‐59	164 197 (68 725/95472)	251
rs6717858	2	165 539 661	COBLL1;SCN2A	C/T	0.400	−0.051	0.008	2.04E‐11	164 197 (68 725/95472)	41
rs7117339	11	93 870 338	PANX1	T/C	0.120	−0.130	0.011	2.26E‐30	164 197 (68 725/95472)	140
rs72754571	15	90 350 888	ANPEP	A/C	0.081	−0.083	0.014	2.47E‐09	164 197 (68 725/95472)	35
rs738409	22	44 324 727	PNPLA3	G/C	0.228	0.269	0.009	1.00E‐200	164 197 (68 725/95472)	893
rs7604422	2	233 509 761	EFHD1	C/A	0.401	−0.053	0.008	1.24E‐12	164 197 (68 725/95472)	44
rs79598313	1	27 284 913	PIGV;GPN2;SYTL1;SLC9A1	T/C	0.023	0.180	0.025	3.37E‐13	164 197 (68 725/95472)	52
rs848559	2	36 694 497	CRIM1	T/A	0.151	−0.061	0.011	7.59E‐09	164 197 (68 725/95472)	31
rs9867368	3	136 147 771	PCCB	A/G	0.198	−0.078	0.009	1.61E‐17	164 197 (68 725/95472)	75
Cirrhosis (European populations‐five cohorts)										
rs12904	1	155 106 697	EFNA1	A/G	0.400	−0.105	0.025	1.70E‐37	77 534 (4829/72705)	18
rs2642438	1	220 970 028	MARC1/A165T	A/G	0.290	−0.094	0.028	1.20E‐43	77 534 (4829/72705)	11
rs429358	19	45 411 941	APOE	C/T	0.150	−0.163	0.039	7.00E‐36	77 534 (4829/72705)	17
rs58542926	19	19 379 549	TM6SF2	T/C	0.070	0.365	0.043	2.80E‐83	77 534 (4829/72705)	72
rs6834314	4	88 213 808	HSD17B13	G/A	0.260	−0.163	0.033	2.80E‐54	77 534 (4829/72705)	24
rs7029757	9	132 566 666	TOR1B	A/G	0.090	−0.163	0.048	1.40E‐12	77 534 (4829/72705)	12
rs738409	22	44 324 727	PNPLA3	G/C	0.230	0.489	0.028	1.00E‐200	77 534 (4829/72705)	305
Cirrhosis (European populations‐FinnGen)										
rs117930290	9	12 196 079	RP11‐74C3.1	C/T	0.023	0.612	0.115	1.00E‐07	218 792 (1931/216861)	28
rs187429064	19	19 380 513	TM6SF2	G/A	0.051	0.412	0.077	9.75E‐08	218 792 (1931/216861)	28
rs73017891	19	22 228 179	AC003973.5	C/G	0.022	0.584	0.118	6.67E‐07	218 792 (1931/216861)	25
rs738409	22	44 324 727	PNPLA3	G/C	0.227	0.461	0.041	1.20E‐29	218 792 (1931/216861)	128
rs74421592	1	213 584 297	RPL31P13	A/G	0.023	0.584	0.115	3.50E‐07	218 792 (1931/216861)	26
rs9992651	4	88 232 510	HSD17B13	A/G	0.210	−0.206	0.041	3.72E‐07	218 792 (1931/216861)	26
Cirrhosis (East Asian populations)										
rs12484700	22	44 327 273	PNPLA3	G/A	0.454	0.219	0.031	1.05E‐12	212 453 (2184/210269)	51
rs3129943	6	32 338 695	TSBP1	G/A	0.364	0.220	0.032	3.63E‐12	212 453 (2184/210269)	48
rs78069066	12	112 337 924	MAPKAPK5	A/G	0.231	−0.278	0.039	1.08E‐12	212 453 (2184/210269)	51

Abbreviations: CHR, chromosome; EA, effect allele; EAF, effect allele frequency; NAFLD, non‐alcoholic fatty liver disease; NEA, non‐effect allele; SE, standard error; SNP, single‐nucleotide polymorphism.

### 2.4 Statistical analysis

GWAS data on outcomes were extracted according to the IVs of exposures. We then harmonized these GWAS data were to make the effect allele (compared to non‐effect allele) was correlated with higher risks of exposures.

We used random‐effects inverse‐variance weighted (IVW) as the primary approach to compute odds ratios (ORs) and 95% confidence intervals (95% CIs). The heterogeneity of SNPs was tested by Cochran's Q test (*P*_heterogeneity_ < 0.05 indicated heterogeneity presence). Furthermore, we applied weighted median, weighted mode, and MR Egger to test whether the main results were robust. We also established scatter plots to visualize these univariable results.

An IV should fulfill three MR assumptions: relevance assumption (the IV is correlated with the exposure), independence assumption (the IV is not related to confounders), and exclusion restriction assumption (the IV influences the outcome only through the exposure in question).^13^, ^14^, ^15^ For the first assumption, we computed the *F*‐statistics by: $F={\text{beta}}^{2}/{\mathrm{se}}^{2}$, where beta and se are the coefficient and standard error of the SNP‐exposure association.^22^ The *F*‐statistics were 33–212, 29–893, 11–305, 25–128, and 48–51 for NAFLD (European populations), cALT (European populations), cirrhosis (European populations‐five cohorts), cirrhosis (European populations‐FinnGen), and cirrhosis (East Asian populations), respectively (Table 2), implying strong IVs were employed (*F*‐statistics > 10 was considered as strong IVs).^22^

Moreover, several sensitivity analyses were conducted to partially test the independence and exclusion restriction assumptions. First, we applied MR Egger intercept test to partially detect horizontal pleiotropy (an IV influences the outcome via other exposures other than the one under investigation). Horizontal pleiotropy is absent if MR Egger intercept was not significantly different from zero (*P*_pleiotropy_ > 0.05).^13^, ^14^, ^15^ Second, we built leave‐one‐out plots to find SNPs significantly influenced the MR estimates. Third, we performed additional MR analyses to investigate whether exposures were associated with some potential confounders. For European populations, the included confounders were overweight, obesity class 1, obesity class 2, obesity class 3, ever versus never smokers, cigarettes consumption (per day), alcohol consumption (per week), hypertension, coronary artery disease, and type 2 diabetes (T2D). For East Asian populations, body mass index (BMI), congestive heart failure, T2D, low‐density lipoprotein cholesterol, high‐density lipoprotein cholesterol, total cholesterol, smoking, diastolic blood pressure, systolic blood pressure, mean arterial pressure, chronic hepatitis B, chronic hepatitis C, and ever/never alcohol drinker were included as the confounders. We obtained these GWAS data from the MR‐Base database (https://gwas.mrcieu.ac.uk/).^15^ Table 1 showed the details of data sources. Fourth, we carried out further MR analyses to assess whether these potential confounders were correlated with outcomes. Fifth, we performed multivariable MR analyses by including exposures and these possible confounders as independent variables.

Furthermore, due to the presence of shared samples in both the GWAS data for exposures and outcomes, there is a possibility of encountering winner's curse and weak instrument bias.^23^, ^24^ In order to partially evaluate the impact of sample overlap, an additional sensitivity analysis was carried out for NAFLD in European populations. This analysis utilized GWAS data that had been conducted after the exclusion of samples that were shared between the datasets. Additionally, since the SNPs used for cirrhosis in East Asian populations included variants close to ALDH2 (rs78069066) and HLA‐region (rs3129943), it was possible that alcohol intake or viral hepatitis affected the MR results. Therefore, an additional sensitivity analysis was performed to detect the effect of each SNP on the MR estimates.

All analyses were performed using the TwoSampleMR package (version 0.5.6) within R (version 4.2.0). Statistical significance was determined based on a two‐sided *p*‐value of less than .05.

## 3 RESULTS

Among European subjects, genetically‐predicted NAFLD had a positive association with liver and intrahepatic bile ducts cancer, with ORs (95% CIs) of 6.62 (3.81, 11.50) (*p* < .001) using IVW, 6.47 (3.47, 12.07) (*p* < .001) using weighted median, and 6.01 (2.96, 12.17) (*p* = .038) using weighted mode (Table 3, Figure S1). However, the positive relationship was insignificant when applying MR Egger, with an OR (95% CI) being 5.71 (0.95, 34.23) (*p* = .308) (Table 3, Figure S1). Heterogeneity was not found by Cochran's Q test (*P*_heterogeneity_ = 0.831) (Table 3). In sensitivity analyses, horizontal pleiotropy was not observed by MR Egger intercept test (*P*_pleiotropy_ = 0.892) (Table 3). The leave‐one‐out plot revealed that no outlier SNP significantly affected the results (Figure S2). NAFLD was unrelated to all included confounders (Table S2). Likewise, most of these confounders were not related to liver and intrahepatic bile ducts cancer except for coronary artery disease (Table S3). NAFLD was still positively associated with liver and intrahepatic bile ducts cancer after adjustments in multivariable MR analyses, with an OR (95% CI) of 1.39 (1.09, 1.78) (*p* = .009) (Table 3). Additionally, the characteristics of NAFLD‐related SNPs after excluding overlapped sample were shown in Table S4. The positive relationship still existed after excluding overlapped sample (Table S5).

**TABLE 3:** MR estimates for the associations of NAFLD with liver and intrahepatic bile ducts cancer in European populations.

Exposure	Outcome	MR method^a^	OR (95% CI)	*p*	*P*_heterogeneity_	MR Egger intercept (SE)	*P*_pleiotropy_
NAFLD	Liver and intrahepatic bile ducts cancer	IVW	6.62 (3.81, 11.50)	<.001	0.831		
NAFLD	Liver and intrahepatic bile ducts cancer	Weighted median	6.47 (3.47, 12.07)	<.001			
NAFLD	Liver and intrahepatic bile ducts cancer	Weighted mode	6.01 (2.96, 12.17)	.038			
NAFLD	Liver and intrahepatic bile ducts cancer	MR Egger	5.71 (0.95, 34.23)	.308		0.037 (0.218)	0.892
NAFLD	Liver and intrahepatic bile ducts cancer	Multivariable MR	1.39 (1.09, 1.78)	.009			

Abbreviations: CI, confidence interval; IVW, inverse‐variance weighted; MR, Mendelian randomization; NAFLD, non‐alcoholic fatty liver disease; OR, odds ratio; SE, standard error.

^a^ Overweight, obesity class 1, obesity class 2, obesity class 3, ever versus never smokers, cigarettes consumption (per day), alcohol consumption (per week), hypertension, coronary artery disease, and type 2 diabetes were adjusted for in multivariable MR analyses.

In addition, genetically‐predicted cALT was positively related to liver and intrahepatic bile ducts cancer in European populations. The ORs (95% CIs) were 2.59 (1.70, 3.94) (*p* < .001) for IVW, 3.22 (1.78, 5.82) (*p* < .001) for weighted median, 4.85 (2.56, 9.19) (*p* < .001) for weighted mode, and 5.20 (2.54, 10.64) (*p* < .001) for MR Egger (Table 4, Figure S3). Heterogeneity was detected using Cochran's Q test (*P*_heterogeneity_ = 0.014) (Table 4). In sensitivity analyses, there was no horizontal pleiotropy in MR Egger intercept test (*P*_pleiotropy_ = 0.667) (Table 4). The leave‐one‐out plot indicated that no outlier SNP significantly influenced the results (Figure S4). There were no associations between cALT and most included confounders except for T2D (Table S2). Most included confounders were not associated with liver and intrahepatic bile ducts cancer except for coronary artery disease (Table S3). The positive relationship between cALT and liver and intrahepatic bile ducts cancer still existed in multivariable MR analyses (OR = 2.13; 95% CI = 1.47–3.43; *p* < .001) (Table 4).

**TABLE 4:** MR estimates for the associations of cALT with liver and intrahepatic bile ducts cancer in European populations.

Exposure	Outcome	MR method^a^	OR (95% CI)	*p*	*P*_heterogeneity_	MR Egger intercept (SE)	*P*_pleiotropy_
cALT	Liver and intrahepatic bile ducts cancer	IVW	2.59 (1.70, 3.94)	<.001	0.014		
cALT	Liver and intrahepatic bile ducts cancer	Weighted median	3.22 (1.78, 5.82)	<.001			
cALT	Liver and intrahepatic bile ducts cancer	Weighted mode	4.85 (2.56, 9.19)	<.001			
cALT	Liver and intrahepatic bile ducts cancer	MR Egger	5.20 (2.54, 10.64)	<.001		−0.077 (0.183)	0.667
cALT	Liver and intrahepatic bile ducts cancer	Multivariable MR	2.13 (1.47, 3.43)	<.001			

Abbreviations: cALT, chronic elevation of alanine aminotransferase; CI, confidence interval; IVW, inverse‐variance weighted; MR, Mendelian randomization; OR, odds ratio; SE, standard error.

^a^ Overweight, obesity class 1, obesity class 2, obesity class 3, ever versus never smokers, cigarettes consumption (per day), alcohol consumption (per week), hypertension, coronary artery disease, and type 2 diabetes were adjusted for in multivariable MR analyses.

Using cirrhosis data that were derived from a GWAS of five European cohorts, a positive relationship was found between genetically‐predicted cirrhosis and liver and intrahepatic bile ducts cancer. The ORs (95% CIs) were 3.38 (2.41, 4.75) (*p* < .001) using IVW, 3.21 (2.19, 4.71) (*p* < .001) using weighted median, 3.11 (2.06, 4.69) (*p* = .002) using weighted mode, and 2.91 (1.55, 5.44) (*p* = .021) using MR Egger (Table 5, Figure S5). No heterogeneity was found by Cochran's Q test (*P*_heterogeneity_ = 0.352) (Table 5). In sensitivity analyses, horizontal pleiotropy was not detected by MR Egger intercept test (*P*_pleiotropy_ = 0.589) (Table 5). The leave‐one‐out plot suggested that no outlier SNP significantly affected the results (Figure S6). Cirrhosis was not related to all included confounders (Table S2). Similarly, most of these confounders were not associated with liver and intrahepatic bile ducts cancer except for coronary artery disease (Table S3). In multivariable MR analyses, cirrhosis was still positively related to liver and intrahepatic bile ducts cancer (OR = 2.43; 95% CI = 1.87–2.96; *p* < .001) (Table 5).

**TABLE 5:** MR estimates for the associations of cirrhosis (five cohorts) with liver and intrahepatic bile ducts cancer in European populations.

Exposure	Outcome	MR method^a^	OR (95% CI)	*p*	*P*_heterogeneity_	MR Egger intercept (SE)	*P*_pleiotropy_
Cirrhosis	Liver and intrahepatic bile ducts cancer	IVW	3.38 (2.41, 4.75)	<.001	0.352		
Cirrhosis	Liver and intrahepatic bile ducts cancer	Weighted median	3.21 (2.19, 4.71)	<.001			
Cirrhosis	Liver and intrahepatic bile ducts cancer	Weighted mode	3.11 (2.06, 4.69)	.002			
Cirrhosis	Liver and intrahepatic bile ducts cancer	MR Egger	2.91 (1.55, 5.44)	.021		0.045 (0.077)	0.589
Cirrhosis	Liver and intrahepatic bile ducts cancer	Multivariable MR	2.43 (1.87, 2.96)	<.001			

Abbreviations: CI, confidence interval; IVW, inverse‐variance weighted; MR, Mendelian randomization; OR, odds ratio; SE, standard error.

^a^ Overweight, obesity class 1, obesity class 2, obesity class 3, ever versus never smokers, cigarettes consumption (per day), alcohol consumption (per week), hypertension, coronary artery disease, and type 2 diabetes were adjusted for in multivariable MR analyses.

Using cirrhosis data that were obtained from FinnGen, we found that genetically‐predicted cirrhosis was positively related to liver and intrahepatic bile ducts cancer, with ORs (95% CIs) of 2.62 (1.20, 5.72) (*p* = .015) for IVW, 3.13 (2.02, 4.84) (*p* < .001) for weighted median, and 2.76 (1.78, 4.27) (*p* = .006) for weighted mode; while MR Egger found an insignificant relationship (OR = 1.28; 95% CI = 0.13–13.14; *p* = .843) (Table 6, Figure S7). Heterogeneity was detected by Cochran's Q test (*P*_heterogeneity_ < 0.001) (Table 6). In sensitivity analyses, horizontal pleiotropy was not observed by MR Egger intercept test (*P*_pleiotropy_ = 0.555) (Table 6). The leave‐one‐out plot indicated that no outlier SNP significantly influenced the results (Figure S8). Cirrhosis was not related to most included confounders except for T2D (Table S2). Likewise, most of these confounders were not related to liver and intrahepatic bile ducts cancer except for coronary artery disease (Table S3). The positive relationship of cirrhosis with liver and intrahepatic bile ducts cancer remained after adjustments in multivariable MR analyses (OR = 2.27; 95% CI = 1.58–3.26; *p* < .001) (Table 6).

**TABLE 6:** MR estimates for the associations of cirrhosis (FinnGen) with liver and intrahepatic bile ducts cancer in European populations.

Exposure	Outcome	MR method^a^	OR (95% CI)	*p*	*P*_heterogeneity_	MR Egger intercept (SE)	*P*_pleiotropy_
Cirrhosis	Liver and intrahepatic bile ducts cancer	IVW	2.62 (1.20, 5.72)	.015	<0.001		
Cirrhosis	Liver and intrahepatic bile ducts cancer	Weighted median	3.13 (2.02, 4.84)	<.001			
Cirrhosis	Liver and intrahepatic bile ducts cancer	Weighted mode	2.76 (1.78, 4.27)	.006			
Cirrhosis	Liver and intrahepatic bile ducts cancer	MR Egger	1.28 (0.13, 13.14)	.843		0.310 (0.482)	0.555
Cirrhosis	Liver and intrahepatic bile ducts cancer	Multivariable MR	2.27 (1.58, 3.26)	<.001			

Abbreviations: CI, confidence interval; IVW, inverse‐variance weighted; MR, Mendelian randomization; OR, odds ratio; SE, standard error.

^a^ Overweight, obesity class 1, obesity class 2, obesity class 3, ever versus never smokers, cigarettes consumption (per day), alcohol consumption (per week), hypertension, coronary artery disease, and type 2 diabetes were adjusted for in multivariable MR analyses.

Among East Asian subjects, genetically‐predicted cirrhosis had a positive association with HCC, with ORs (95% CIs) being 2.12 (1.78, 2.52) (*p* < .001) using IVW, 2.15 (1.68, 2.75) (*p* < .001) using weighted median, and 2.17 (1.62, 2.90) (*p* = .035) using weighted mode (Table 7, Figure S9). Nonetheless, an insignificant relationship was found by MR Egger, with an OR (95% CI) of 2.40 (0.49, 11.80) (*p* = .477) (Table 7, Figure S9). Heterogeneity was not observed by Cochran's Q test (*P*_heterogeneity_ = 0.929) (Table 7). In sensitivity analyses, no horizontal pleiotropy was found using MR Egger intercept test (*P*_pleiotropy_ = 0.903) (Table 7). The leave‐one‐out plot indicated that no SNP significantly influenced the results (Figure S10). Cirrhosis was not correlated with most included confounders except for BMI and chronic hepatitis B (Table S2). Similarly, most of these confounders were not related to HCC except for low‐density lipoprotein cholesterol and chronic hepatitis B (Table S3). Cirrhosis was still positively related to HCC after adjustments in multivariable MR analyses (OR = 1.79; 95% CI = 1.56–2.05; *p* < .001) (Table 7). Moreover, when only using the SNP not related to ALDH2 and HLA region (rs12484700), cirrhosis was still positively correlated with HCC with an OR (95% CI) of 2.02 (1.50–2.72) (*p* < .001) (Table S6).

**TABLE 7:** MR estimates for the association between cirrhosis and hepatocellular carcinoma in East Asian populations.

Exposure	Outcome	MR method^a^	OR (95% CI)	*p*	*P*_heterogeneity_	MR Egger intercept (SE)	*P*_pleiotropy_
Cirrhosis	Hepatocellular carcinoma	IVW	2.12 (1.78, 2.52)	<.001	0.929		
Cirrhosis	Hepatocellular carcinoma	Weighted median	2.15 (1.68, 2.75)	<.001			
Cirrhosis	Hepatocellular carcinoma	Weighted mode	2.17 (1.62, 2.90)	.035			
Cirrhosis	Hepatocellular carcinoma	MR Egger	2.40 (0.49, 11.80)	.477		−0.029 (0.192)	0.903
Cirrhosis	Hepatocellular carcinoma	Multivariable MR	1.79 (1.56, 2.05)	<.001			

Abbreviations: CI, confidence interval; IVW, inverse‐variance weighted; MR, Mendelian randomization; OR, odds ratio; SE, standard error.

^a^ Body mass index, smoking, total cholesterol, low‐density lipoprotein cholesterol, high‐density lipoprotein cholesterol, diastolic blood pressure, systolic blood pressure, mean arterial pressure, congestive heart failure, type 2 diabetes, chronic hepatitis B, chronic hepatitis C, and ever/never alcohol drinker were adjusted for in multivariable MR analyses.

## 4 DISCUSSION

The current MR research found positive associations of genetically‐predicted NAFLD, cALT, and cirrhosis with liver and intrahepatic bile ducts cancer in European populations. The positive relationship was also observed between genetically‐predicted cirrhosis and HCC in East Asian populations. These results were consistent and robust when using different MR methods except for the null associations found in some MR Egger analyses. Furthermore, horizontal pleiotropy was not found using MR Egger intercept test. The exposures were not related to most included confounders except for T2D in European subjects and BMI and chronic hepatitis B in East Asian populations. Likewise, there were no associations between most included confounders and liver cancer except for coronary artery disease in European people and low‐density lipoprotein cholesterol and chronic hepatitis B in East Asian populations. Additionally, these positive associations were consistent after adjustments in multivariable MR analyses. Further MR analyses produced consistent results for NAFLD (European populations) after excluding overlapped sample.

Consistent with our study, previous observational studies reported the positive relationships of NAFLD and cirrhosis with liver cancer. For NAFLD, a systematic review and meta‐analysis of 103 observational studies including 948 217 subjects showed that NAFLD was positively associated with HCC risk with a hazard ratio (HR) (95% CI) being 1.88 (1.46, 2.42) (*p* < .01).^10^ Another systematic review and meta‐analysis of 19 studies involving 168 571 subjects without cirrhosis suggested that, compared to patients with other liver diseases (hepatitis B virus, hepatitis C virus, alcoholic liver disease, etc.), those with non‐alcoholic steatohepatitis (NASH) were at a greater risk of HCC (OR = 2.61; 95% CI = 1.27–5.35; *p* = .009).^25^ In addition, a systematic review and meta‐analysis of 61 studies showed that patients with HCC but no cirrhosis accounted for 38.5% for all NAFLD patients, which was higher than that for hepatitis B virus (21.7%), hepatitis C virus (6.4%), and alcohol‐associated liver disease (9.1%).^26^ A European cohort study involving 2.741 NASH cases and 66 209 non‐NASH cases with liver transplantation found that, NASH patients had a greater proportion of HCC patients than non‐NASH patients (39.1 vs. 28.9%, *p* < .001).^27^ A Japanese review of 34 studies implied that HCC ranked the first for cancer incidence and mortality in NAFLD/NASH patients.^12^

For cirrhosis, a systematic review and meta‐analysis of 18 studies including 470 404 patients indicated that the incidence rates of HCC were significantly higher in cirrhosis patients (3.78 per 100 person‐years; 95% CI = 2.47–5.78) than in subjects without cirrhosis (0.03 per 100 person‐years; 95% CI = 0.01–0.07).^11^ Another systematic review and meta‐analysis of 16 publications with 13 576 patients revealed that subjects with primary biliary cirrhosis had a significantly higher HCC risk than those without (pooled risk ratio = 18.80; 95% CI = 10.81–26.79).^28^ A Taiwan study of 2568 pairs of cirrhosis patients and controls revealed that HCC incidence was higher in complicated cirrhosis patients than in uncomplicated cirrhosis subjects (HR = 1.23; 95% CI = 1.10–1.37; *p* < .001).^29^

To our knowledge, there were no MR studies being conducted to explore whether NAFLD and cirrhosis were causally related to liver cancer. Existing MR studies assessed the potential causal associations of NAFLD with cardiovascular disease,^30^, ^31^, ^32^, ^33^, ^34^ diabetes,^30^, ^35^, ^36^ obesity,^36^ insulin resistance,^37^ psoriasis,^38^ and kidney function.^39^ A MR study investigated the potential causal relationship between primary biliary cirrhosis and hypothyroidism.^40^ These MR studies suggested that NAFLD and cirrhosis were risk factors for the diseases/disorders of interest.^30^, ^31^, ^32^, ^33^, ^34^, ^35^, ^36^, ^37^, ^38^, ^39^, ^40^ Consistent results were observed in prior observational studies.^41^, ^42^ These results implied that NAFLD and cirrhosis may interact with some other factors and then increase liver cancer risk. However, in sensitivity analyses, most confounders were not simultaneously correlated with exposures and outcomes (except for chronic hepatitis B in East Asian populations). In addition, all positive associations still existed in multivariable MR analyses after adjusting for some confounders. These results indicated that the exposures may have independent effects on liver cancer.

There were several explanations for the positive relationships of NAFLD and cirrhosis with liver cancer.^43^ For NAFLD, hepatic adipose tissue may facilitate the secretion of some inflammatory cytokines (like tumor necrosis factor‐α and interleukin 6), reduce adiponectin levels, and then cause insulin resistance.^43^ Insulin resistance could inhibit fatty acids oxidation, thereby increasing intracellular fatty acids levels that may lead to DNA damage and genetic mutations.^43^ Meanwhile, insulin resistance could stimulate the release of insulin‐like growth factor 1 and insulin receptor substrate 1, which may influence cell proliferation and apoptosis.^43^ For cirrhosis, recurrent cycles of hepatocellular death and compensatory regeneration, along with continual cell growth and proliferation that favor tumor formation, were the main mechanisms of cirrhosis‐induced liver cancer.^44^

For a MR study to be valid, assumptions of relevance, independence, and exclusion restriction should be met.^13^, ^14^, ^15^ In this study, since the F‐statistic of all used SNPs was larger than 10, the relevance assumption was satisfied. For the independence and exclusion restriction assumptions, no horizontal pleiotropy was observed using MR Egger intercept test. However, in European populations, some SNPs used for NAFLD and cirrhosis were located in the same genes, suggesting the presence of pleiotropic effect. Nonetheless, the pleiotropic effect can be categorized as horizontal pleiotropy (where the genetic variant influences the outcome through another factor other than the one of interest) and vertical pleiotropy (where the genetic variant initially affects the target factor under investigation and subsequently impacts other elements, eventually influencing the outcome).^13^, ^14^, ^15^ The existence of horizontal pleiotropy suggested a breach in the assumptions of independence and exclusion restriction.^13^, ^14^, ^15^ On the other hand, vertical pleiotropy is acceptable as it demonstrated that a factor has a downstream effect on an outcome.^13^, ^14^, ^15^ In the current research, the pleiotropic effect was more likely to be vertical pleiotropy because many studies indicated a NAFLD → cirrhosis → liver cancer pathway.^2^ Nevertheless, further MR investigations are needed to explore the independent impact of NAFLD on liver cancer when GWASs identify genetic variants exclusively associated with NAFLD.

Additionally, in East Asian populations, the SNPs used for cirrhosis included variants related to ALDH2 (rs78069066) and HLA‐region (rs3129943), indicating that alcohol intake or viral hepatitis may influence the MR results. Meanwhile, in sensitivity analyses, chronic hepatitis B was positively related to the exposure and outcome simultaneously, suggesting a violation of independence and exclusion restriction assumptions. However, in multivariable analyses, the positive association between cirrhosis and HCC still existed after adjusting for many potential confounders including chronic hepatitis B, chronic hepatitis C, and ever/never alcohol drinker. Furthermore, when only using the other cirrhosis‐related SNP (rs12484700) in sensitivity analysis, cirrhosis was still positively related to HCC. These findings implied that the present MR study may not violate the two assumptions.

Although this study investigated the potential causal relationships of NAFLD and cirrhosis with liver cancer in European and East Asian populations, there are certain limitations to consider. First, the number of liver cancer cases within the European population subset was relatively small. To validate our results, it is necessary to conduct MR studies using larger GWAS datasets that encompass a greater number of liver cancer patients in future. Second, the inability to conduct subgroup analyses based on significant factors like age, sex, and tumor stages is attributed to the utilization of summarized GWAS data. Third, it is important to note that the data utilized in this study exclusively pertained to European and East Asian populations. Therefore, applying these findings to other ethnic groups should be approached with careful consideration. Fourth, the results of MR‐Egger were insignificant for NAFLD (European populations), cirrhosis (European populations‐FinnGen), and cirrhosis (East Asian populations), which were inconsistent with the results of other MR methods. The main reason is that the estimates of MR‐Egger are particularly sensitive to outlying and influential datapoints.^45^ Meanwhile, the results of MR‐Egger were reported to be less accurate compared to other methods, especially when the SNP‐exposure relationships had similar effect sizes.^45^ These factors can result in MR‐Egger having a low power to detect a causal association.^45^ Therefore, further MR studies with more exposure‐related SNPs and more patients are needed.

Fifth, the GWAS data on exposures and outcomes were overlapped to some degree, which may introduce winner's curse and weak instrument bias.^23^, ^24^ Nevertheless, for NAFLD (European populations), we found consistent results using GWAS data after excluding overlapped sample, suggesting the results might not be biased by sample overlap. For cirrhosis (European‐FinnGen and East Asian populations), although we cannot perform similar sensitivity analyses because all subjects were derived from the same databases, a previous empirical investigation indicated that the impact of sample overlap in such cases may not be substantial.^24^ Specifically, for cirrhosis (European‐FinnGen and East Asian populations), a one‐sample MR framework was employed. Within this framework, the associations between genetic variants and exposure as well as the associations between the same genetic variants and the outcome were derived from the identical populations. These associations were computed through a meta‐analysis of both the discovery and replication cohorts.^24^ Under such circumstances, the MR estimates would be deflated by winner's curse with exposure and inflated by winner's curse with outcome and weak instrument bias.^24^ As a result, these competing biases approximately canceled out, and there were no significant differences in the MR estimates between this scenario and a two‐sample scenario.^24^ Nevertheless, this does not imply that our analyses are exempt from the winner's curse or the potential bias arising from weak instruments. Hence, it is imperative that forthcoming MR studies be conducted when GWAS performed on different populations that do not overlap become accessible.

In general, this MR investigation revealed that within European subjects, genetically‐predicted NAFLD, cALT levels, and cirrhosis exhibited positive relationships with liver and intrahepatic bile ducts cancer. Within East Asian subjects, genetically‐predicted cirrhosis displayed a positive association with HCC. These findings serve as additional supportive evidence for the favorable associations identified in earlier observational studies. Subsequent MR investigations should be undertaken when GWAS involving more liver cancer patients from non‐overlapped populations become available.

## AUTHOR CONTRIBUTIONS

**Yunyang Deng:** Conceptualization (lead); data curation (lead); formal analysis (lead); investigation (lead); methodology (lead); resources (lead); visualization (lead); writing – original draft (lead); writing – review and editing (equal). **Junjie Huang:** Project administration (equal); supervision (equal); writing – review and editing (equal). **Martin CS Wong:** Project administration (equal); supervision (equal); writing – review and editing (equal).

## CONFLICT OF INTEREST STATEMENT

The authors have stated explicitly that there are no conflicts of interest in connection with this article.

## ETHICS STATEMENT

All related studies used in this study were approved by appropriate ethics review boards. Written informed consents were obtained from all participants. This study did not exceed the scope of the original ethics committee approvals.

## Supporting information


**Table S1.** STROBE checklist of the current study.
**Table S2.** MR estimates for the associations of NAFLD and cirrhosis with potential confounders in European and East Asian populations.
**Table S3.** MR estimates for the associations of potential confounders with liver cancer in European and East Asian populations.
**Table S4.** Characteristics of SNPs associated with NAFLD in European populations (after excluding overlapped sample).
**Table S5.** MR estimates for the association of NAFLD with liver and intrahepatic bile ducts cancer in European populations (after excluding overlapped sample).
**Table S6.** MR estimates for the association between cirrhosis and hepatocellular carcinoma in East Asian populations using individual SNPs.
**Figure S1.** Scatter plot for the association between NAFLD and liver and intrahepatic bile ducts cancer in European populations. This plot showed the effect size of NAFLD on liver and intrahepatic bile ducts cancer using four MR univariable methods. The X‐axis is the effect of SNP on NAFLD (beta ± 95% confidence interval). The Y‐axis is the effect of SNP on liver and intrahepatic bile ducts cancer (beta ± 95% confidence interval). The line slope is the effect size.
**Figure S2.** Leave‐one‐out plot for the association between NAFLD and liver and intrahepatic bile ducts cancer in European populations. This plot showed the effect size of NAFLD on liver and intrahepatic bile ducts cancer when excluding one SNP at a time from all used SNPs. The X‐axis is the effect size (beta ± 95% confidence interval). The Y‐axis is the ID of SNP.
**Figure S3.** Scatter plot for the association between cALT and liver and intrahepatic bile ducts cancer in European populations. This plot showed the effect size of cALT on liver and intrahepatic bile ducts cancer using four MR univariable methods. The X‐axis is the effect of SNP on cALT (beta ± 95% confidence interval). The Y‐axis is the effect of SNP on liver and intrahepatic bile ducts cancer (beta ± 95% confidence interval). The line slope is the effect size.
**Figure S4.** Leave‐one‐out plot for the association between cALT and liver and intrahepatic bile ducts cancer in European populations. This plot showed the effect size of cALT on liver and intrahepatic bile ducts cancer when excluding one SNP at a time from all used SNPs. The X‐axis is the effect size (beta ± 95% confidence interval). The Y‐axis is the ID of SNP.
**Figure S5.** Scatter plot for the association between cirrhosis (five cohorts) and liver and intrahepatic bile ducts cancer in European populations. This plot showed the effect size of cirrhosis (five cohorts) on liver and intrahepatic bile ducts cancer using four MR univariable methods. The X‐axis is the effect of SNP on cirrhosis (five cohorts) (beta ± 95% confidence interval). The Y‐axis is the effect of SNP on liver and intrahepatic bile ducts cancer (beta ± 95% confidence interval). The line slope is the effect size.
**Figure S6.** Leave‐one‐out plot for the association between cirrhosis (five cohorts) and liver and intrahepatic bile ducts cancer in European populations. This plot showed the effect size of cirrhosis (five cohorts) on liver and intrahepatic bile ducts cancer when excluding one SNP at a time from all used SNPs. The X‐axis is the effect size (beta ± 95% confidence interval). The Y‐axis is the ID of SNP.
**Figure S7.** Scatter plot for the association between cirrhosis (FinnGen) and liver and intrahepatic bile ducts cancer in European populations. This plot showed the effect size of cirrhosis (FinnGen) on liver and intrahepatic bile ducts cancer using four MR univariable methods. The X‐axis is the effect of SNP on cirrhosis (FinnGen) (beta ± 95% confidence interval). The Y‐axis is the effect of SNP on liver and intrahepatic bile ducts cancer (beta ± 95% confidence interval). The line slope is the effect size.
**Figure S8.** Leave‐one‐out plot for the association between cirrhosis (FinnGen) and liver and intrahepatic bile ducts cancer in European populations. This plot showed the effect size of cirrhosis (FinnGen) on liver and intrahepatic bile ducts cancer when excluding one SNP at a time from all used SNPs. The X‐axis is the effect size (beta ± 95% confidence interval). The Y‐axis is the ID of SNP.
**Figure S9.** Scatter plot for the association between cirrhosis and hepatocellular carcinoma in East Asian populations. This plot showed the effect size of cirrhosis on hepatocellular carcinoma using four MR univariable methods. The X‐axis is the effect of SNP on cirrhosis (beta ± 95% confidence interval). The Y‐axis is the effect of SNP on hepatocellular carcinoma (beta ± 95% confidence interval). The line slope is the effect size.
**Figure S10.** Leave‐one‐out plot for the association between cirrhosis and hepatocellular carcinoma in East Asian populations. This plot showed the effect size of cirrhosis on hepatocellular carcinoma when excluding one SNP at a time from all used SNPs. The X‐axis is the effect size (beta ± 95% confidence interval). The Y‐axis is the ID of SNP.

## Data Availability

All data used in this study are publicly available without any access restrictions. One can also contact the correspondence author if he or she would like to assess them.
