# Supplementary material for: Associations of non‐alcoholic fatty liver disease and cirrhosis with liver cancer in European and East Asian populations: A Mendelian randomization study
Source: Cancer Rep (Hoboken). 2023 Oct 16;7(1):e1913. doi: 10.1002/cnr2.1913 (PMC10809194; doi:10.1002/cnr2.1913)

**Title:** Associations of non-alcoholic fatty liver disease and cirrhosis with liver cancer in European and East Asian populations: a Mendelian randomization study

**Authors:** Yunyang Deng, MPhil; Junjie Huang, Ph.D.; Martin Chi Sang Wong, M.D.

**Correspondence author:** Professor Martin Chi Sang Wong; Email address: wong_martin@cuhk.edu.hk; Tel: +852 2252 8782.

| **Contents** | **Pages** |
|---|---|
| Supplementary table 1. STROBE checklist of the current study | 3-5 |
| Supplementary table 2. MR estimates for the associations of NAFLD and cirrhosis with potential confounders in European and East Asian populations | 6-8 |
| Supplementary table 3. MR estimates for the associations of potential confounders with liver cancer in European and East Asian populations | 9-10 |
| Supplementary table 4. Characteristics of SNPs associated with NAFLD in European populations (after excluding overlapped sample) | 11 |
| Supplementary table 5. MR estimates for the association of NAFLD with liver and intrahepatic bile ducts cancer in European populations (after excluding overlapped sample) | 12 |
| Supplementary table 6. MR estimates for the association between cirrhosis and hepatocellular carcinoma in East Asian populations using individual SNPs | 13 |
| Supplementary figure 1. Scatter plot for the association between NAFLD and liver and intrahepatic bile ducts cancer in European populations | 14 |
| Supplementary figure 2. Leave-one-out plot for the association between NAFLD and liver and intrahepatic bile ducts cancer in European populations | 15 |
| Supplementary figure 3. Scatter plot for the association between cALT and liver and intrahepatic bile ducts cancer in European populations | 16 |
| Supplementary figure 4. Leave-one-out plot for the association between cALT and liver and intrahepatic bile ducts cancer in European populations | 17 |
| Supplementary figure 5. Scatter plot for the association between cirrhosis (five cohorts) and liver and intrahepatic bile ducts cancer in European populations | 18 |

| **Contents** | **Pages** |
|---|---|
| Supplementary figure 6. Leave-one-out plot for the association between cirrhosis (five cohorts) and liver and intrahepatic bile ducts cancer in European populations | 19 |
| Supplementary figure 7. Scatter plot for the association between cirrhosis (FinnGen) and liver and intrahepatic bile ducts cancer in European populations | 20 |
| Supplementary figure 8. Leave-one-out plot for the association between cirrhosis (FinnGen) and liver and intrahepatic bile ducts cancer in European populations | 21 |
| Supplementary figure 9. Scatter plot for the association between cirrhosis and hepatocellular carcinoma in East Asian populations | 22 |
| Supplementary figure 10. Leave-one-out plot for the association between cirrhosis and hepatocellular carcinoma in East Asian populations | 23 |

**Supplementary table 1. STROBE checklist of the current study**

| | Item No. | Recommendation | Page No. |
|---|---|---|---|
| **Title and abstract** | 1 | (*a*) Indicate the study's design with a commonly used term in the title or the abstract | 1-4 |
| | | (*b*) Provide in the abstract an informative and balanced summary of what was done and what was found | 3-4 |
| **Introduction** | | | |
| Background/rationale | 2 | Explain the scientific background and rationale for the investigation being reported | 5-6 |
| Objectives | 3 | State specific objectives, including any prespecified hypotheses | 6 |
| **Methods** | | | |
| Study design | 4 | Present key elements of study design early in the paper | 7 |
| Setting | 5 | Describe the setting, locations, and relevant dates, including periods of recruitment, exposure, follow-up, and data collection | 7-12 |
| Participants | 6 | (*a*) *Cohort study*-Give the eligibility criteria, and the sources and methods of selection of participants. Describe methods of follow-up | 7-12 |
| | | *Case-control study*-Give the eligibility criteria, and the sources and methods of case ascertainment and control selection. Give the rationale for the choice of cases and controls | |
| | | *Cross-sectional study*-Give the eligibility criteria, and the sources and methods of selection of participants | |
| | | (*b*) *Cohort study*-For matched studies, give matching criteria and number of exposed and unexposed | NA |
| | | *Case-control study*-For matched studies, give matching criteria and the number of controls per case | |
| Variables | 7 | Clearly define all outcomes, exposures, predictors, potential confounders, and effect modifiers. Give diagnostic criteria, if applicable | 7-12 |
| Data sources/ measurement | 8 | For each variable of interest, give sources of data and details of methods of assessment (measurement). Describe comparability of assessment methods if there is more than one group | 7-12 |

| | Item No. | Recommendation | Page No. |
|---|---|---|---|
| Bias | 9 | Describe any efforts to address potential sources of bias | 12-15 |
| Study size | 10 | Explain how the study size was arrived at | 7-12 |
| Quantitative variables | 11 | Explain how quantitative variables were handled in the analyses. If applicable, describe which groupings were chosen and why | 12-15 |
| Statistical methods | 12 | (*a*) Describe all statistical methods, including those used to control for confounding | 12-15 |
| | | (*b*) Describe any methods used to examine subgroups and interactions | 12-15 |
| | | (*c*) Explain how missing data were addressed | 7-12 |
| | | (*d*) *Cohort study*-If applicable, explain how loss to follow-up was addressed | NA |
| | | *Case-control study*-If applicable, explain how matching of cases and controls was addressed | |
| | | *Cross-sectional study*-If applicable, describe analytical methods taking account of sampling strategy | |
| | | (*e*) Describe any sensitivity analyses | 12-15 |
| **Results** | | | |
| Participants | 13 [a] | (a) Report numbers of individuals at each stage of study-eg numbers potentially eligible, examined for eligibility, confirmed eligible, included in the study, completing follow-up, and analyzed | 7-12 |
| | | (b) Give reasons for non-participation at each stage | NA |
| | | (c) Consider use of a flow diagram | NA |
| Descriptive data | 14 [a] | (a) Give characteristics of study participants (eg demographic, clinical, social) and information on exposures and potential confounders | 7-12 |
| | | (b) Indicate number of participants with missing data for each variable of interest | NA |
| | | (c) *Cohort study*-Summarise follow-up time (eg, average and total amount) | NA |
| Outcome data | 15 [a] | Cohort study-Report numbers of outcome events or summary measures over time | NA |
| | | *Case-control study*-Report numbers in each exposure category, or summary measures of exposure | NA |
| | | *Cross-sectional study*-Report numbers of outcome events or summary measures | 7-12 |

| | Item No. | Recommendation | Page No. |
|---|---|---|---|
| Main results | 16 | (*a*) Give unadjusted estimates and, if applicable, confounder-adjusted estimates and their precision (eg, 95% confidence interval). Make clear which confounders were adjusted for and why they were included | 16-19; Tables 3-7 |
| | | (*b*) Report category boundaries when continuous variables were categorized | NA |
| | | (*c*) If relevant, consider translating estimates of relative risk into absolute risk for a meaningful time period | NA |
| Other analyses | 17 | Report other analyses done-eg analyses of subgroups and interactions, and sensitivity analyses | 16-19; Table 3-7; STs 2-6; SFs 1-10 |
| **Discussion** | | | |
| Key results | 18 | Summarize key results with reference to study objectives | 20 |
| Limitations | 19 | Discuss limitations of the study, taking into account sources of potential bias or imprecision. Discuss both direction and magnitude of any potential bias | 24-26 |
| Interpretation | 20 | Give a cautious overall interpretation of results considering objectives, limitations, multiplicity of analyses, results from similar studies, and other relevant evidence | 20-26 |
| Generalizability | 21 | Discuss the generalizability (external validity) of the study results | 25 |
| **Other information** | | | |
| Funding | 22 | Give the source of funding and the role of the funders for the present study and, if applicable, for the original study on which the present article is based | 2 |

STROBE, strengthening the reporting of observational studies in epidemiology; NA, not applicable; ST, supplementary table; SF, supplementary figure. [a] Give information separately for cases and controls in case-control studies and, if applicable, for exposed and unexposed groups in cohort and cross-sectional studies.

**Supplementary table 2. MR estimates for the associations of NAFLD and cirrhosis with potential confounders in European and East Asian populations**

| Exposure | Outcome [a] | No of SNPs | Method | OR (95% CI) | P | $P_{heterogeneity}$ | $P_{pleiotropy}$ |
|---|---|---|---|---|---|---|---|
| European populations | | | | | | | |
| NAFLD | Overweight | 1 | WR | 0.99 (0.88, 1.11) | 0.869 | NA | NA |
| NAFLD | Obesity class 1 | 1 | WR | 1.00 (0.85, 1.19) | 0.953 | NA | NA |
| NAFLD | Obesity class 2 | 1 | WR | 0.84 (0.63, 1.13) | 0.253 | NA | NA |
| NAFLD | Obesity class 3 | 1 | WR | 0.76 (0.45, 1.28) | 0.305 | NA | NA |
| NAFLD | Ever vs. never smokers | 1 | WR | 0.96 (0.82, 1.11) | 0.561 | NA | NA |
| NAFLD | Cigarettes consumption (per day) | 3 | IVW | 1.00 (0.92, 1.10) | 0.955 | 0.003 | 0.407 |
| NAFLD | Alcohol consumption (per week) | 3 | IVW | 0.99 (0.97, 1.01) | 0.584 | 0.081 | 0.500 |
| NAFLD | Hypertension | 3 | IVW | 1.00 (1.00, 1.00) | 0.087 | 0.313 | 0.371 |
| NAFLD | Coronary artery disease | 3 | IVW | 0.86 (0.68, 1.09) | 0.211 | <0.001 | 0.229 |
| European populations | | | | | | | |
| cALT | Overweight | 35 | IVW | 0.98 (0.93, 1.03) | 0.457 | 0.008 | 0.957 |
| cALT | Obesity class 1 | 35 | IVW | 0.97 (0.91, 1.04) | 0.400 | 0.033 | 0.673 |
| cALT | Obesity class 2 | 35 | IVW | 0.98 (0.87, 1.10) | 0.722 | 0.006 | 0.832 |
| cALT | Obesity class 3 | 35 | IVW | 0.98 (0.83, 1.16) | 0.835 | 0.510 | 0.680 |
| cALT | Ever vs. never smokers | 36 | IVW | 1.04 (0.97, 1.10) | 0.289 | 0.121 | 0.069 |
| cALT | Cigarettes consumption (per day) | 41 | IVW | 1.04 (1.00, 1.08) | 0.057 | <0.001 | 0.346 |
| cALT | Alcohol consumption (per week) | 41 | IVW | 0.99 (0.97, 1.01) | 0.372 | <0.001 | 0.247 |
| cALT | Hypertension | 43 | IVW | 1.00 (1.00, 1.00) | 0.788 | 0.966 | 0.187 |
| cALT | Coronary artery disease | 41 | IVW | 1.07 (0.98, 1.17) | 0.149 | <0.001 | 0.001 |
| cALT | Type 2 diabetes | 31 | IVW | 1.22 (1.04, 1.43) | 0.015 | <0.001 | 0.671 |

| Exposure | Outcome [a] | No of SNPs | Method | OR (95% CI) | P | $P_{heterogeneity}$ | $P_{pleiotropy}$ |
|---|---|---|---|---|---|---|---|
| European populations (cirrhosis-five cohorts) | | | | | | | |
| Cirrhosis | Overweight | 8 | IVW | 1.00 (0.95, 1.05) | 0.926 | 0.050 | 0.977 |
| Cirrhosis | Obesity class 1 | 8 | IVW | 0.99 (0.92, 1.08) | 0.890 | 0.003 | 0.714 |
| Cirrhosis | Obesity class 2 | 8 | IVW | 1.01 (0.90, 1.14) | 0.846 | 0.005 | 0.944 |
| Cirrhosis | Obesity class 3 | 8 | IVW | 1.02 (0.89, 1.17) | 0.766 | 0.556 | 0.940 |
| Cirrhosis | Ever vs never smokers | 8 | IVW | 0.99 (0.94, 1.04) | 0.630 | 0.298 | 0.160 |
| Cirrhosis | Cigarettes consumption (per day) | 10 | IVW | 1.00 (0.97, 1.03) | 0.971 | 0.004 | 0.476 |
| Cirrhosis | Alcohol consumption (per week) | 10 | IVW | 0.99 (0.98, 1.00) | 0.212 | 0.002 | 0.757 |
| Cirrhosis | Hypertension | 10 | IVW | 1.00 (1.00, 1.00) | 0.175 | 0.780 | 0.119 |
| Cirrhosis | Coronary artery disease | 10 | IVW | 0.97 (0.88, 1.06) | 0.505 | <0.001 | 0.398 |
| Cirrhosis | Type 2 diabetes | 7 | IVW | 1.06 (0.97, 1.16) | 0.201 | 0.145 | 0.281 |
| European populations (cirrhosis-FinnGen) | | | | | | | |
| Cirrhosis | Overweight | 3 | IVW | 0.97 (0.92, 1.02) | 0.222 | 0.191 | 0.320 |
| Cirrhosis | Obesity class 1 | 3 | IVW | 0.94 (0.87, 1.02) | 0.165 | 0.124 | 0.349 |
| Cirrhosis | Obesity class 2 | 2 | IVW | 0.95 (0.74, 1.22) | 0.683 | 0.005 | NA |
| Cirrhosis | Obesity class 3 | 3 | IVW | 0.99 (0.84, 1.17) | 0.908 | 0.365 | 0.494 |
| Cirrhosis | Ever vs never smokers | 3 | IVW | 0.99 (0.88, 1.12) | 0.882 | 0.017 | 0.344 |
| Cirrhosis | Cigarettes consumption (per day) | 6 | IVW | 0.99 (0.96, 1.01) | 0.227 | 0.165 | 0.321 |
| Cirrhosis | Alcohol consumption (per week) | 6 | IVW | 1.00 (0.99, 1.00) | 0.185 | 0.485 | 0.496 |
| Cirrhosis | Hypertension | 6 | IVW | 1.00 (1.00, 1.00) | 0.081 | 0.741 | 0.842 |
| Cirrhosis | Coronary artery disease | 5 | IVW | 0.97 (0.94, 1.00) | 0.082 | 0.904 | 0.723 |
| Cirrhosis | Type 2 diabetes | 2 | IVW | 1.18 (1.01, 1.37) | 0.040 | 0.600 | NA |
| East Asian populations | | | | | | | |

| Exposure | Outcome [a] | No of SNPs | Method | OR (95% CI) | P | $P_{heterogeneity}$ | $P_{pleiotropy}$ |
|---|---|---|---|---|---|---|---|
| Cirrhosis | Body mass index | 2 | IVW | 0.97 (0.94, 0.99) | 0.003 | 0.844 | NA |
| Cirrhosis | Smoking | 2 | IVW | 1.00 (0.99, 1.00) | 0.353 | 0.480 | NA |
| Cirrhosis | Total cholesterol | 2 | IVW | 0.95 (0.88, 1.02) | 0.146 | 0.004 | NA |
| Cirrhosis | Low-density lipoprotein cholesterol | 2 | IVW | 1.00 (0.97, 1.04) | 0.894 | 0.921 | NA |
| Cirrhosis | High-density lipoprotein cholesterol | 2 | IVW | 0.99 (0.92, 1.06) | 0.764 | 0.039 | NA |
| Cirrhosis | Diastolic blood pressure | 2 | IVW | 0.99 (0.96, 1.01) | 0.225 | 0.920 | NA |
| Cirrhosis | Systolic blood pressure | 2 | IVW | 0.99 (0.97, 1.02) | 0.466 | 0.577 | NA |
| Cirrhosis | Mean arterial pressure | 2 | IVW | 0.99 (0.96, 1.01) | 0.246 | 0.732 | NA |
| Cirrhosis | Congestive heart failure | 3 | IVW | 1.02 (0.92, 1.13) | 0.769 | 0.186 | 0.379 |
| Cirrhosis | Type 2 diabetes | 3 | IVW | 1.08 (0.95, 1.24) | 0.238 | <0.001 | 0.667 |
| Cirrhosis | Chronic hepatitis B | 3 | IVW | 1.89 (1.25, 2.84) | 0.002 | 0.015 | 0.843 |
| Cirrhosis | Chronic hepatitis C | 3 | IVW | 1.11 (0.83, 1.50) | 0.480 | <0.001 | 0.448 |
| Cirrhosis | Ever/never alcohol drinker | 3 | IVW | 4.06 (0.33, 50.34) | 0.275 | <0.001 | 0.064 |

MR, Mendelian randomization; NAFLD, non-alcoholic fatty liver disease; SNP, single-nucleotide polymorphism; OR, odds ratio; CI, confidence interval; cALT, chronic elevation of alanine aminotransferase; WR, Wald ratio; NA, not applicable; IVW, inverse-variance weighted.

[a]: Type 2 diabetes was not included as an outcome for NAFLD (European populations) since there was no SNP met the instrumental variable criteria.

**Supplementary table 3. MR estimates for the associations of potential confounders with liver cancer in European and East Asian populations**

| Exposure [a] | Outcome | No of SNPs | Method | OR (95% CI) | P | $P_{heterogeneity}$ | $P_{pleiotropy}$ |
|---|---|---|---|---|---|---|---|
| European populations | | | | | | | |
| Overweight | Liver and intrahepatic bile ducts cancer | 14 | IVW | 1.24 (0.68, 2.28) | 0.480 | 0.974 | 0.650 |
| Obesity class 1 | Liver and intrahepatic bile ducts cancer | 17 | IVW | 1.35 (0.91, 2.01) | 0.135 | 0.974 | 0.680 |
| Obesity class 2 | Liver and intrahepatic bile ducts cancer | 11 | IVW | 1.10 (0.80, 1.52) | 0.562 | 0.728 | 0.570 |
| Obesity class 3 | Liver and intrahepatic bile ducts cancer | 2 | IVW | 1.24 (0.67, 2.28) | 0.489 | 0.115 | NA |
| Ever vs. never smokers | Liver and intrahepatic bile ducts cancer | 1 | WR | 0.79 (0.11, 5.50) | 0.808 | NA | NA |
| Cigarettes consumption (per day) | Liver and intrahepatic bile ducts cancer | 22 | IVW | 1.34 (0.73, 2.46) | 0.346 | 0.481 | 0.284 |
| Alcohol consumption (per week) | Liver and intrahepatic bile ducts cancer | 34 | IVW | 2.31 (0.26, 20.31) | 0.449 | 0.189 | 0.592 |
| Hypertension | Liver and intrahepatic bile ducts cancer | 1 | WR | 6.56E+70 (2.28E-122, 1.89E+263) | 0.471 | NA | NA |
| Coronary artery disease | Liver and intrahepatic bile ducts cancer | 39 | IVW | 0.71 (0.50, 0.99) | 0.046 | 0.872 | 0.243 |
| Type 2 diabetes | Liver and intrahepatic bile ducts cancer | 25 | IVW | 1.23 (0.81, 1.87) | 0.327 | 0.070 | 0.804 |
| East Asian populations | | | | | | | |
| Body mass index | Hepatocellular carcinoma | 65 | IVW | 0.92 (0.68, 1.24) | 0.583 | 0.565 | 0.942 |
| Smoking | Hepatocellular carcinoma | 7 | IVW | 0.28 (0.01, 7.55) | 0.453 | 0.762 | 0.930 |
| Total cholesterol | Hepatocellular carcinoma | 43 | IVW | 0.90 (0.64, 1.27) | 0.560 | 0.028 | 0.788 |
| Low-density lipoprotein cholesterol | Hepatocellular carcinoma | 27 | IVW | 0.62 (0.43, 0.91) | 0.013 | 0.006 | 0.678 |

| Exposure [a] | Outcome | No of SNPs | Method | OR (95% CI) | P | $P_{heterogeneity}$ | $P_{pleiotropy}$ |
|---|---|---|---|---|---|---|---|
| High-density lipoprotein cholesterol | Hepatocellular carcinoma | 48 | IVW | 1.09 (0.90, 1.32) | 0.391 | 0.001 | 0.839 |
| Diastolic blood pressure | Hepatocellular carcinoma | 18 | IVW | 0.97 (0.42, 2.21) | 0.938 | 0.003 | 0.008 |
| Systolic blood pressure | Hepatocellular carcinoma | 22 | IVW | 0.93 (0.48, 1.78) | 0.824 | 0.004 | 0.008 |
| Mean arterial pressure | Hepatocellular carcinoma | 30 | IVW | 0.81 (0.46, 1.41) | 0.448 | 0.007 | 0.010 |
| Congestive heart failure | Hepatocellular carcinoma | 1 | WR | 0.56 (0.27, 1.19) | 0.133 | NA | NA |
| Type 2 diabetes | Hepatocellular carcinoma | 101 | IVW | 0.91 (0.82, 1.01) | 0.065 | 0.005 | 0.632 |
| Chronic hepatitis B | Hepatocellular carcinoma | 5 | IVW | 1.32 (1.20, 1.45) | <0.001 | 0.219 | 0.726 |
| Chronic hepatitis C | Hepatocellular carcinoma | 3 | IVW | 2.89 (2.21, 3.79) | <0.001 | 0.266 | 0.352 |
| Ever/never alcohol drinker | Hepatocellular carcinoma | 3 | IVW | 1.20 (0.95, 1.51) | 0.131 | 0.001 | 0.464 |

MR, Mendelian randomization; NAFLD, non-alcoholic fatty liver disease; SNP, single-nucleotide polymorphism; OR, odds ratio; CI, confidence interval; WR, Wald ratio; NA, not applicable; IVW, inverse-variance weighted.

[a]: We selected SNPs with a more relaxed threshold ($P<1\times10^{-6}$) as instrumental variables for ever vs. never smokers (European populations) and smoking (East Asian populations), since limited SNPs met the conventional threshold ($5\times10^{-8}$) for them.

**Supplementary table 4. Characteristics of SNPs associated with NAFLD in European populations (after excluding overlapped sample)**

| SNPs | CHR | Position | Gene | EA/NEA | EAF | Beta | SE | Sample size (cases/controls) | Explained variance | F |
|---|---|---|---|---|---|---|---|---|---|---|
| After excluding overlapped sample from FinnGen | | | | | | | | | | |
| rs73001065 | 19 | 19460541 | TM6SF2 | C/G | 0.065 | 0.324 | 0.054 | 601,715 (7,783/593,932) | 0.77% | 35 |
| rs429358 | 19 | 45411941 | APOE | T/C | 0.932 | 0.126 | 0.039 | 601,715 (7,783/593,932) | 0.42% | 11 |
| rs3747207 | 22 | 44324855 | PNPLA3 | A/G | 0.222 | 0.287 | 0.033 | 601,715 (7,783/593,932) | 1.12% | 74 |

SNP, single-nucleotide polymorphism; NAFLD, non-alcoholic fatty liver disease; CHR, chromosome; EA, effect allele; NEA, non-effect allele; EAF, effect allele frequency; SE, standard error; TM6SF2, transmembrane 6 superfamily member 2; APOE, apolipoprotein E; PNPLA3, patatin like phospholipase domain containing 3.

**Supplementary table 5. MR estimates for the association of NAFLD with liver and intrahepatic bile ducts cancer in European populations (after excluding overlapped sample)**

| MR methods | OR (95% CI) | P | $P_{heterogeneity}$ | MR Egger intercept (SE) | $P_{pleiotropy}$ |
|---|---|---|---|---|---|
| After excluding overlapped sample from FinnGen | | | | | |
| IVW | 6.59 (4.17, 10.41) | <0.001 | 0.832 | | |
| Weighted median | 6.28 (3.35, 11.79) | <0.001 | | | |
| Weighted mode | 6.05 (3.15, 11.65) | 0.033 | | | |
| MR Egger | 6.83 (1.83, 25.56) | 0.043 | | -0.010 (0.163) | 0.963 |

MR, Mendelian randomization; NAFLD, non-alcoholic fatty liver disease; OR, odds ratio; CI, confidence interval; SE, standard error; IVW, inverse-variance weighted.

**Supplementary table 6. MR estimates for the association between cirrhosis and hepatocellular carcinoma in East Asian populations using individual SNPs**

| **Exposure** | **Outcome** | **SNP** | **OR (95% CI)** | **P** |
|---|---|---|---|---|
| Cirrhosis | Hepatocellular carcinoma | rs12484700 | 2.02 (1.50, 2.72) | <0.001 |
| Cirrhosis | Hepatocellular carcinoma | rs3129943 | 2.18 (1.61, 2.96) | <0.001 |
| Cirrhosis | Hepatocellular carcinoma | rs78069066 | 2.16 (1.61, 2.88) | <0.001 |
| Cirrhosis | Hepatocellular carcinoma | All three SNP/IVW | 2.12 (1.78, 2.52) | <0.001 |

MR, Mendelian randomization; SNP, single-nucleotide polymorphism; OR, odds ratio; CI, confidence interval; IVW, inverse-variance weighted.

**Supplementary figure 1. Scatter plot for the association between NAFLD and liver and intrahepatic bile ducts cancer in European populations**

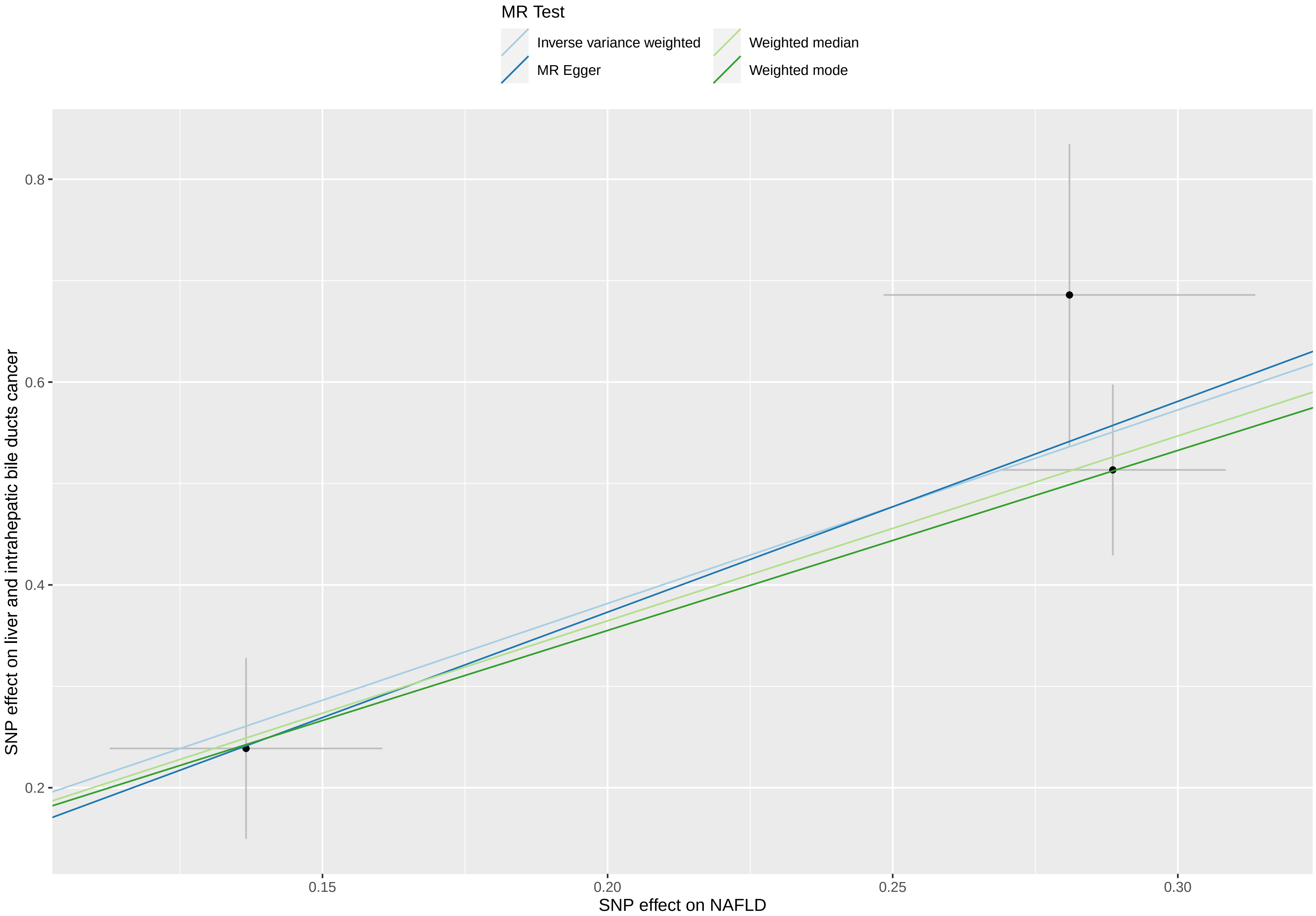

**Supplementary figure 2. Leave-one-out plot for the association between NAFLD and liver and intrahepatic bile ducts cancer in European populations**

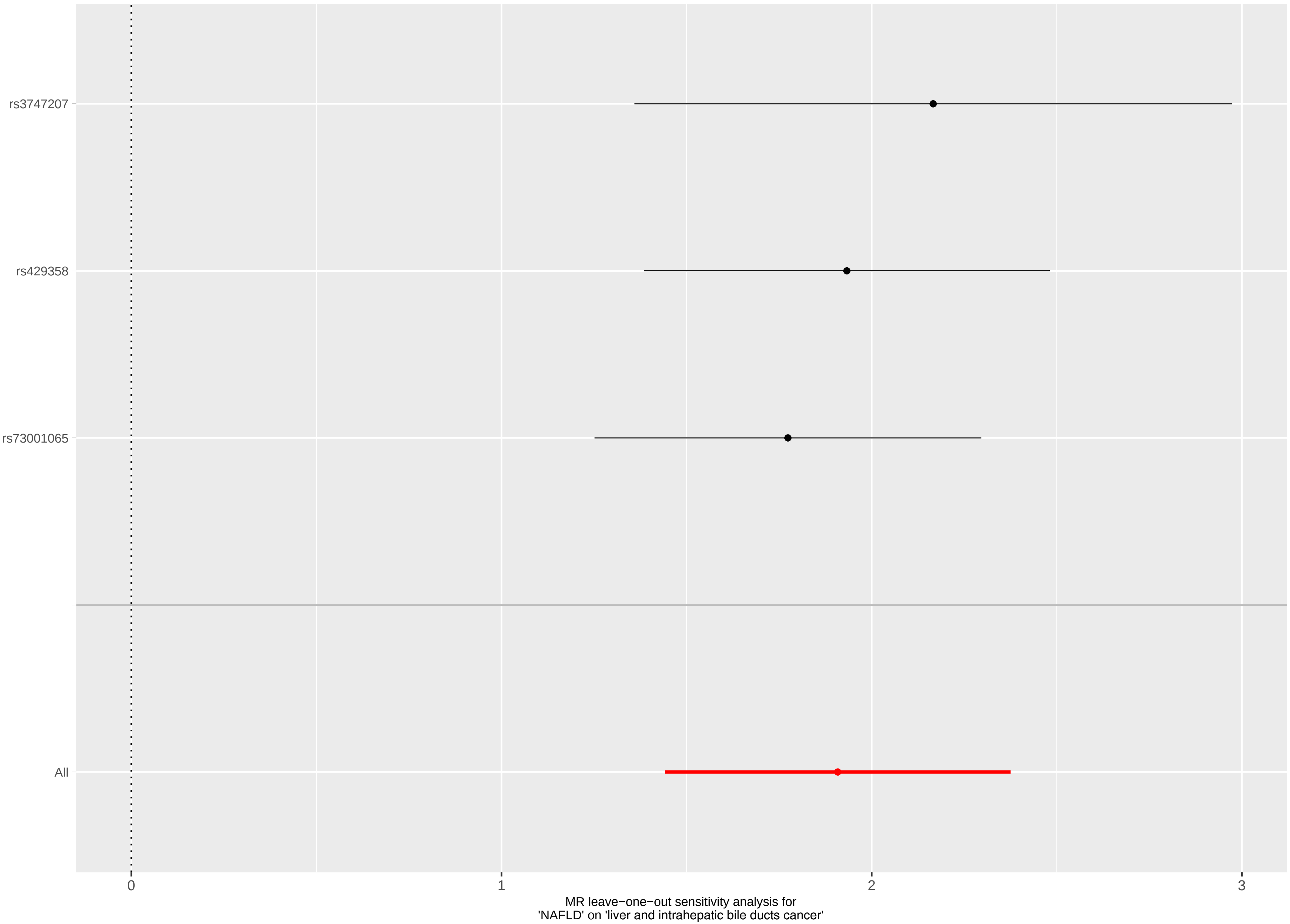

**Supplementary figure 3. Scatter plot for the association between cALT and liver and intrahepatic bile ducts cancer in European populations**

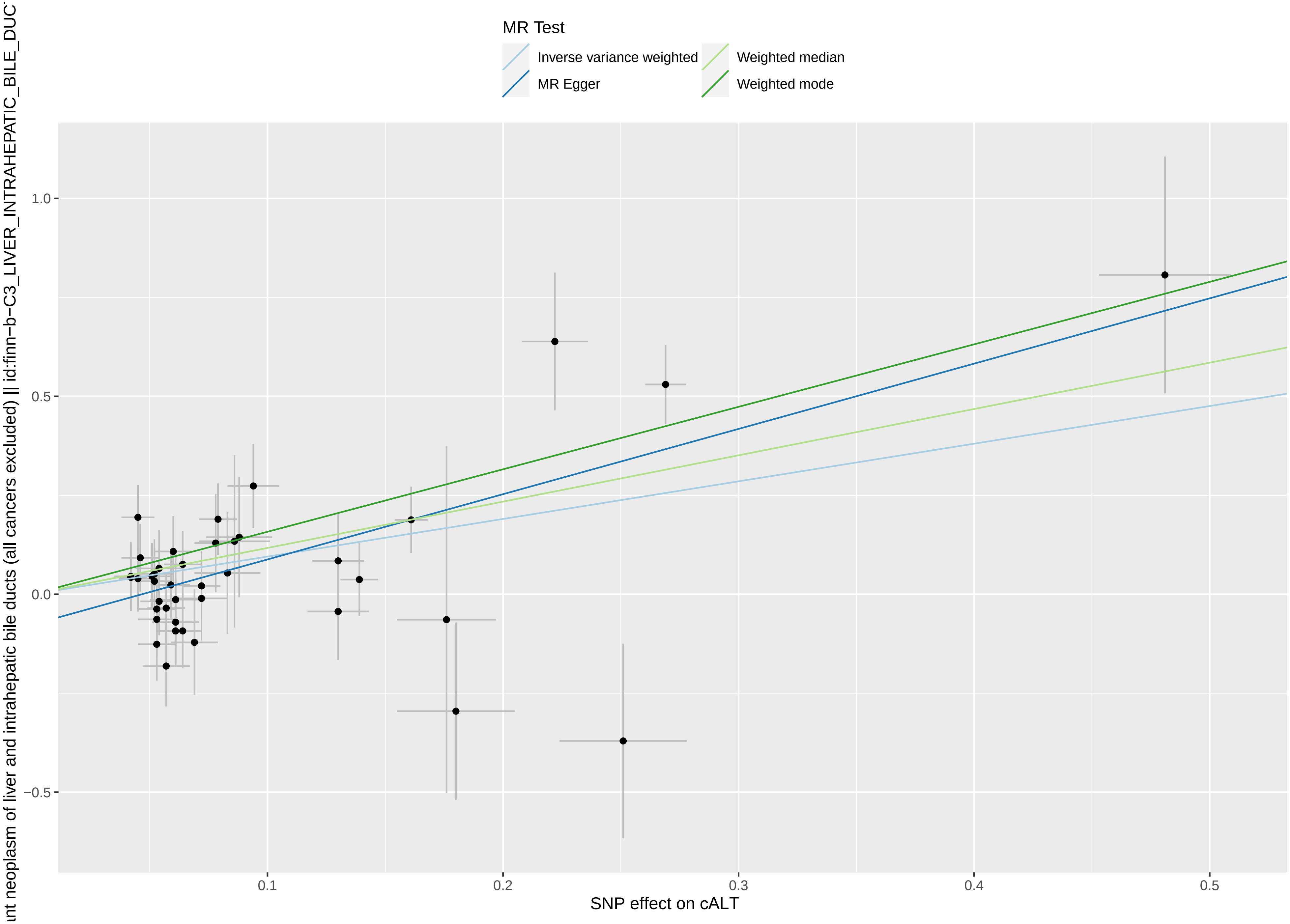

**Supplementary figure 4. Leave-one-out plot for the association between cALT and liver and intrahepatic bile ducts cancer in European populations**

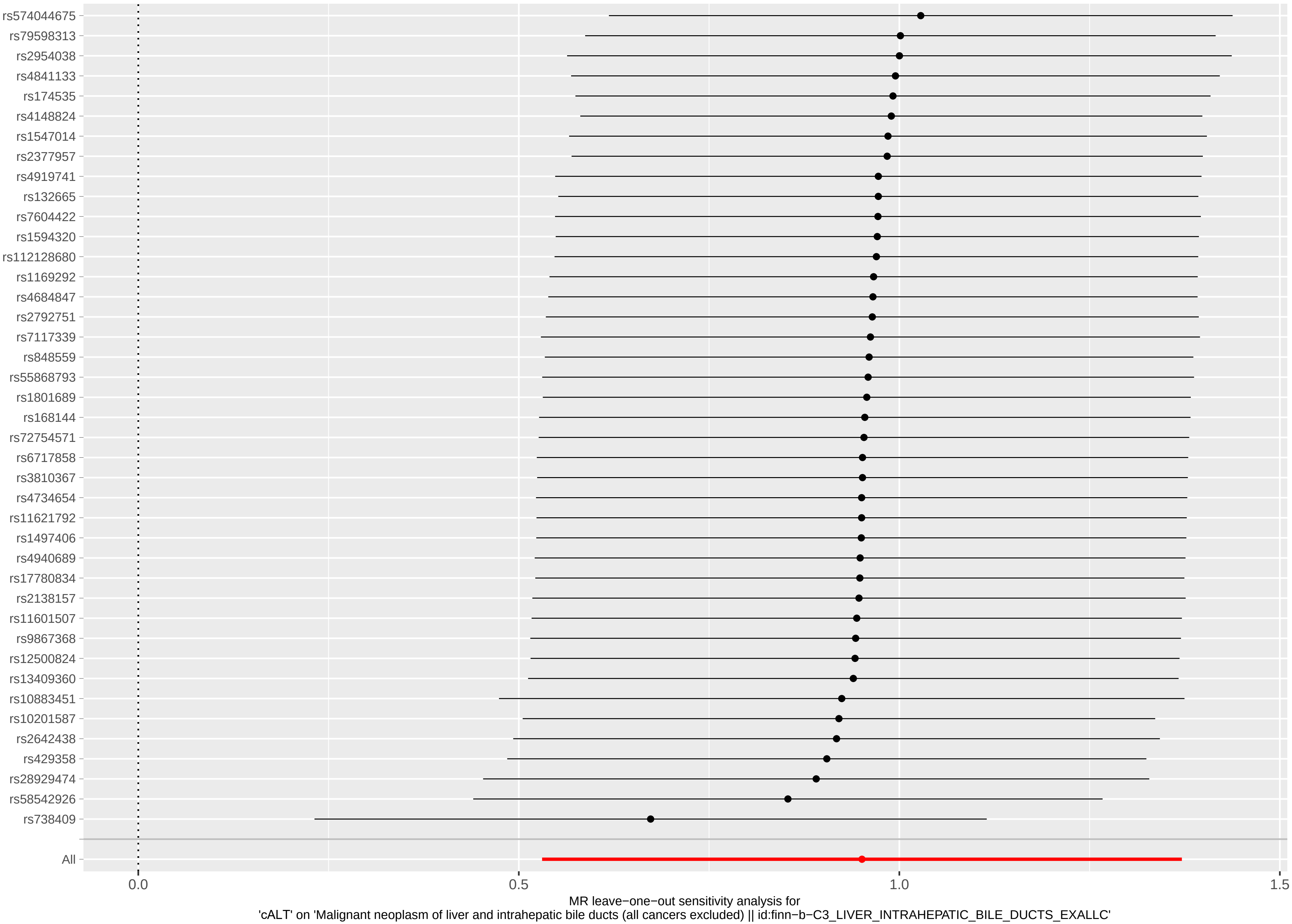

**Supplementary figure 5. Scatter plot for the association between cirrhosis (five cohorts) and liver and intrahepatic bile ducts cancer in European populations**

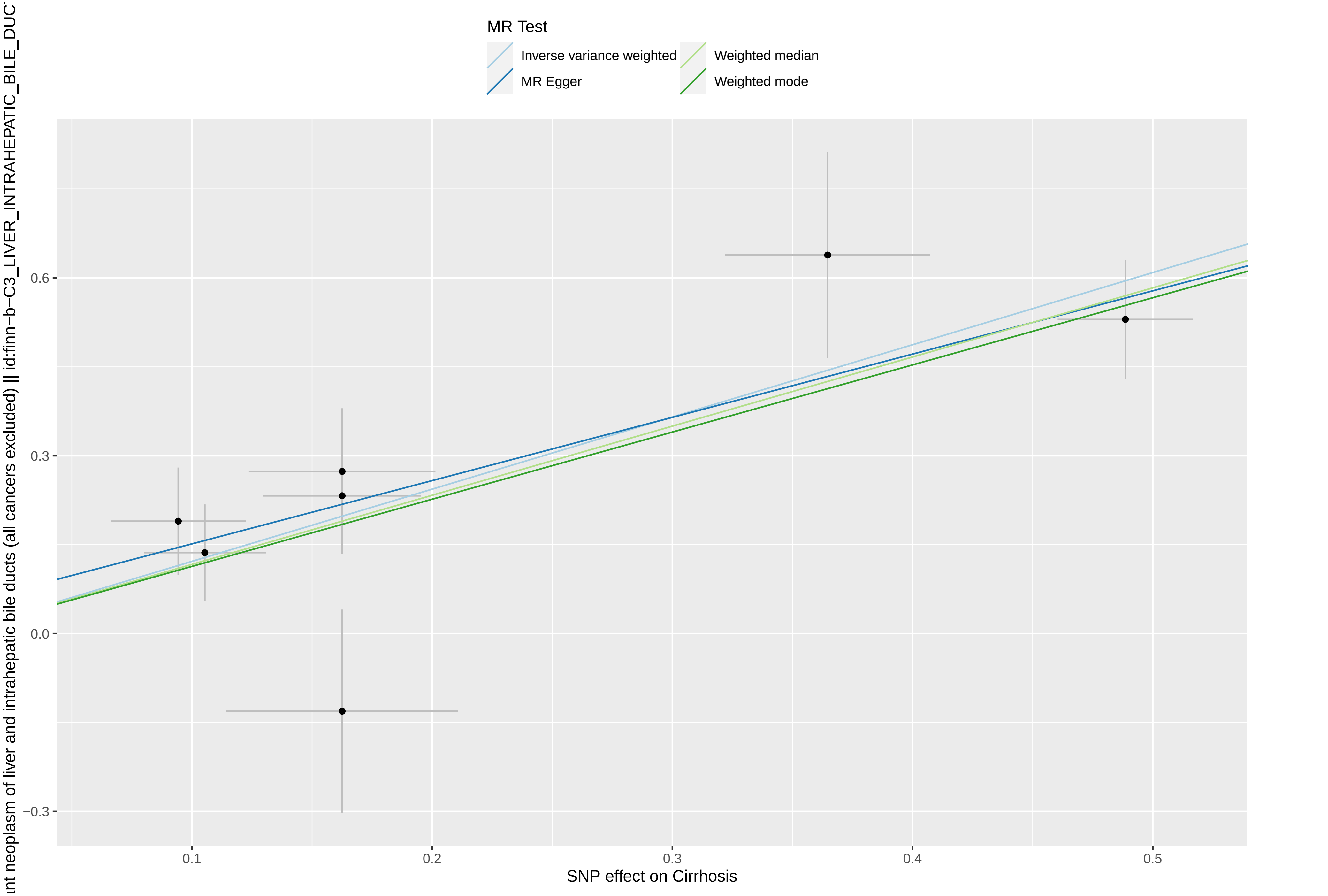

**Supplementary figure 6. Leave-one-out plot for the association between cirrhosis (five cohorts) and liver and intrahepatic bile ducts cancer in European populations**

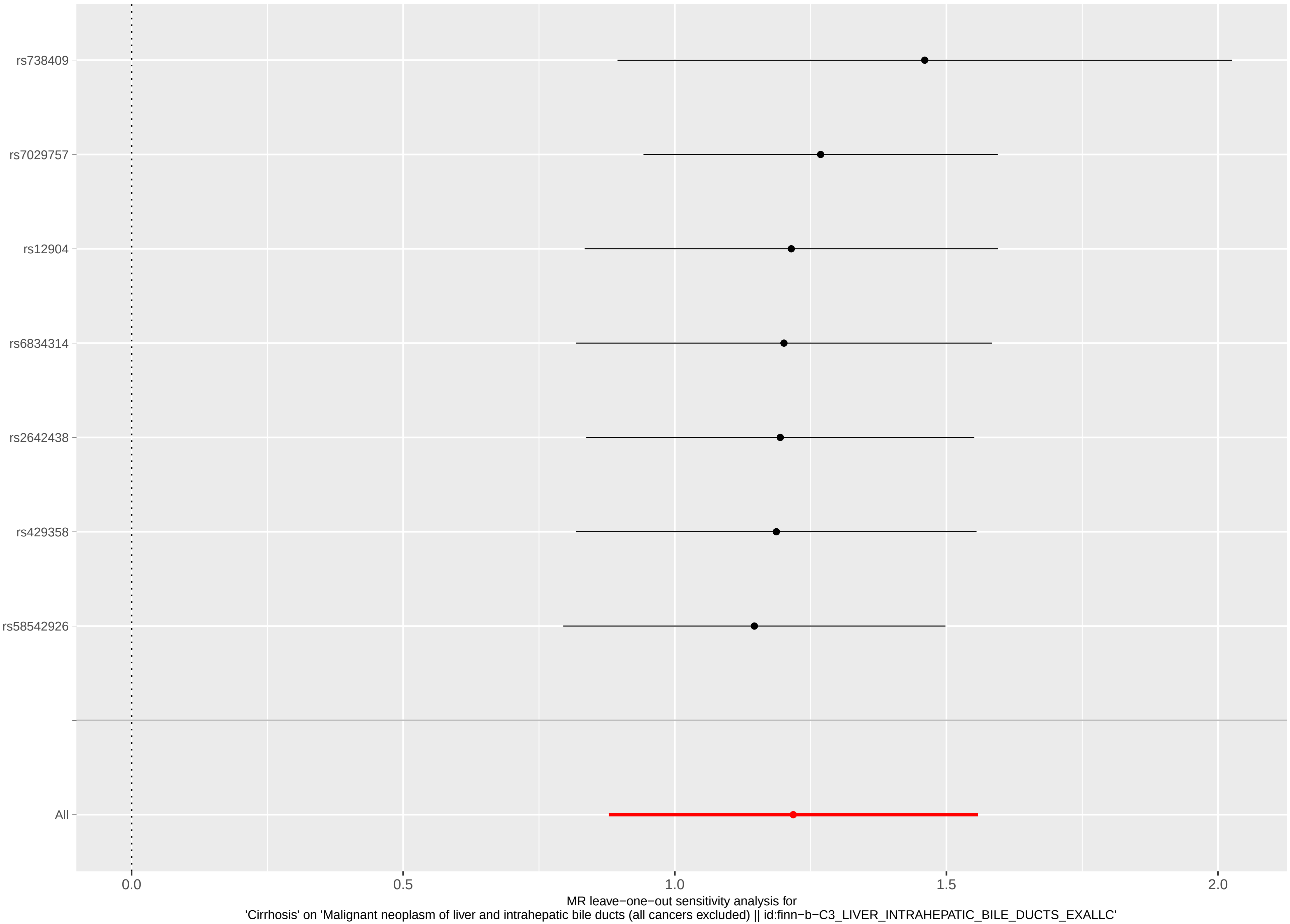

**Supplementary figure 7. Scatter plot for the association between cirrhosis (FinnGen) and liver and intrahepatic bile ducts cancer in European populations**

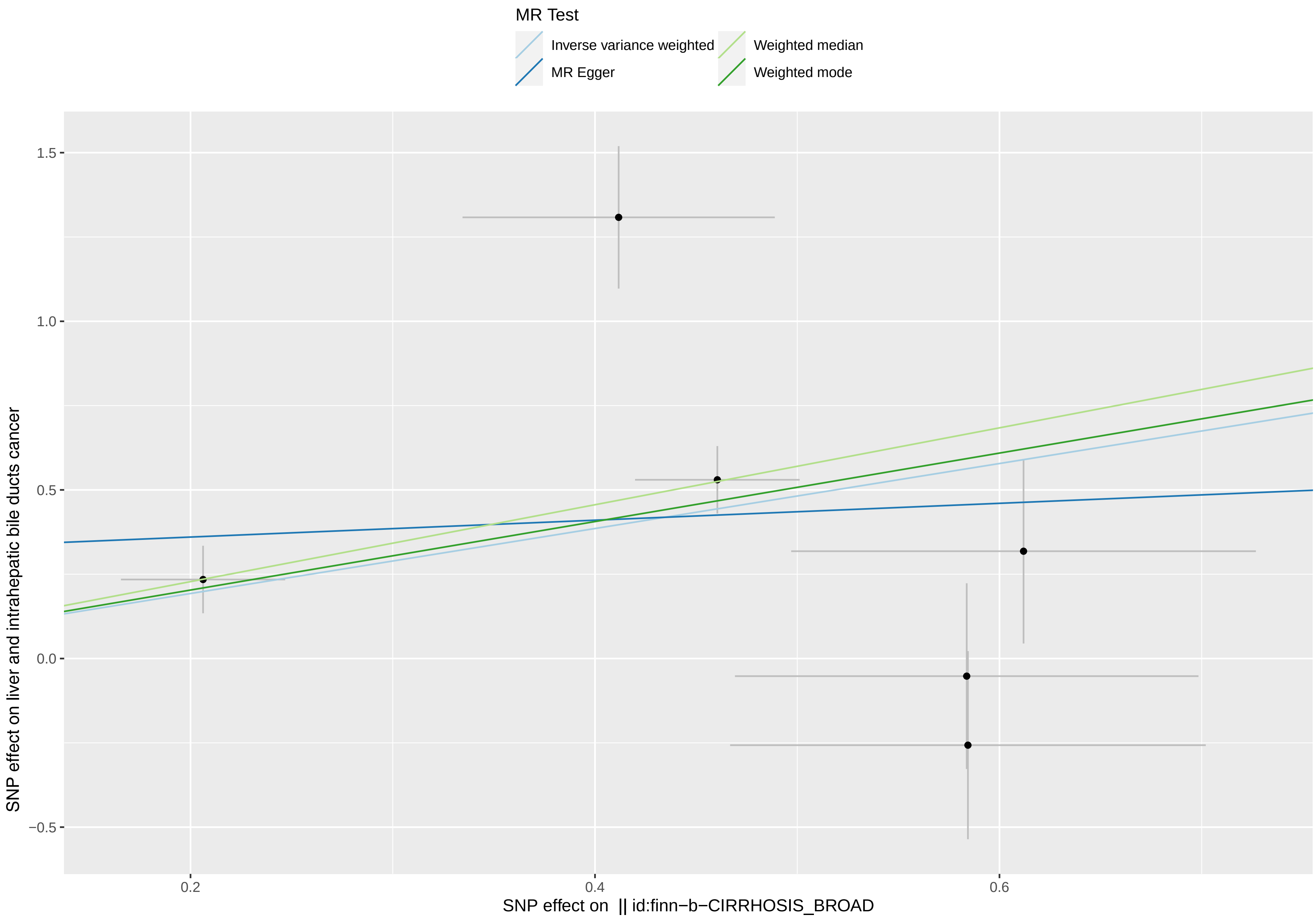

**Supplementary figure 8. Leave-one-out plot for the association between cirrhosis (FinnGen) and liver and intrahepatic bile ducts cancer in European populations**

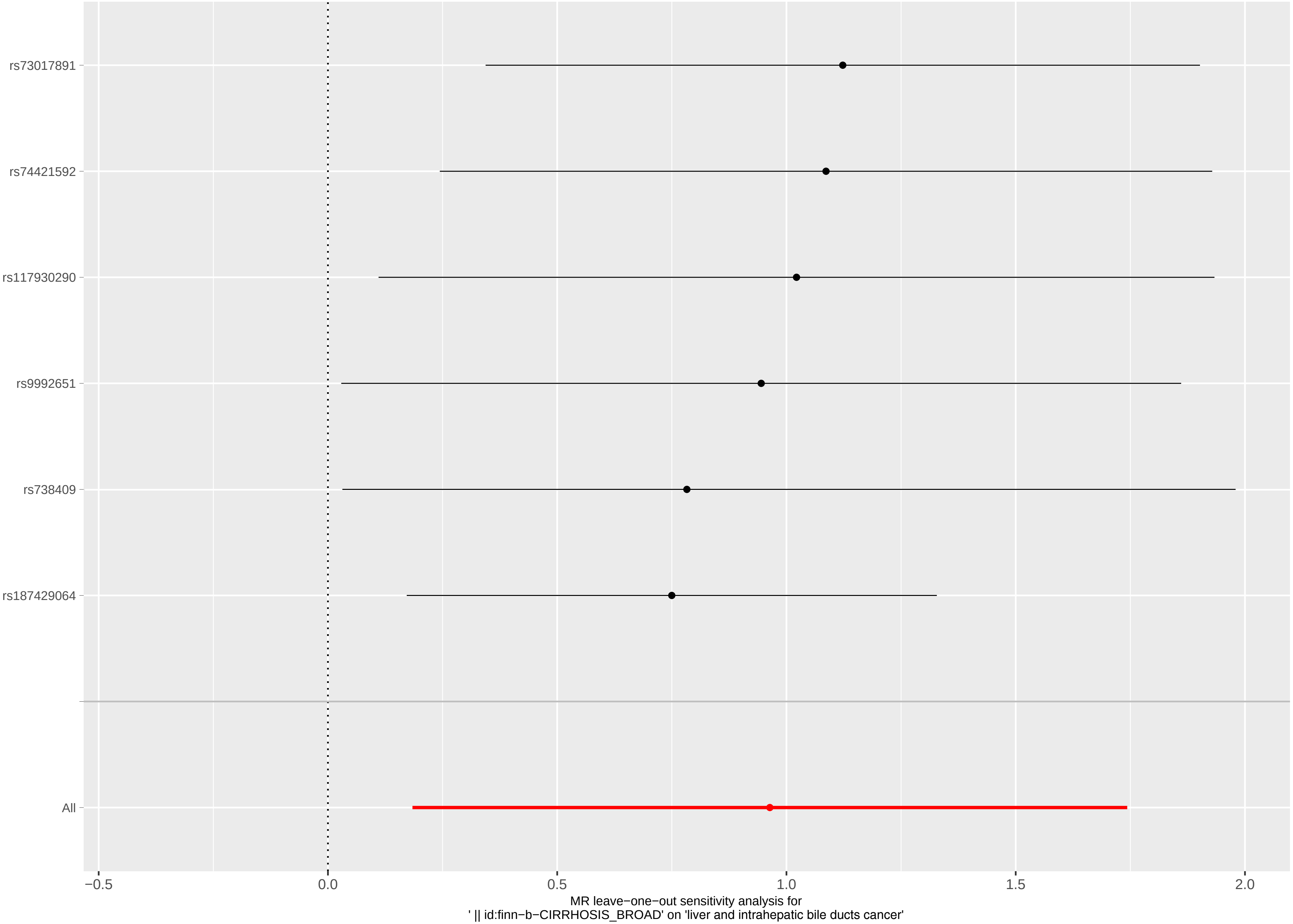

**Supplementary figure 9. Scatter plot for the association between cirrhosis and hepatocellular carcinoma in East Asian populations**

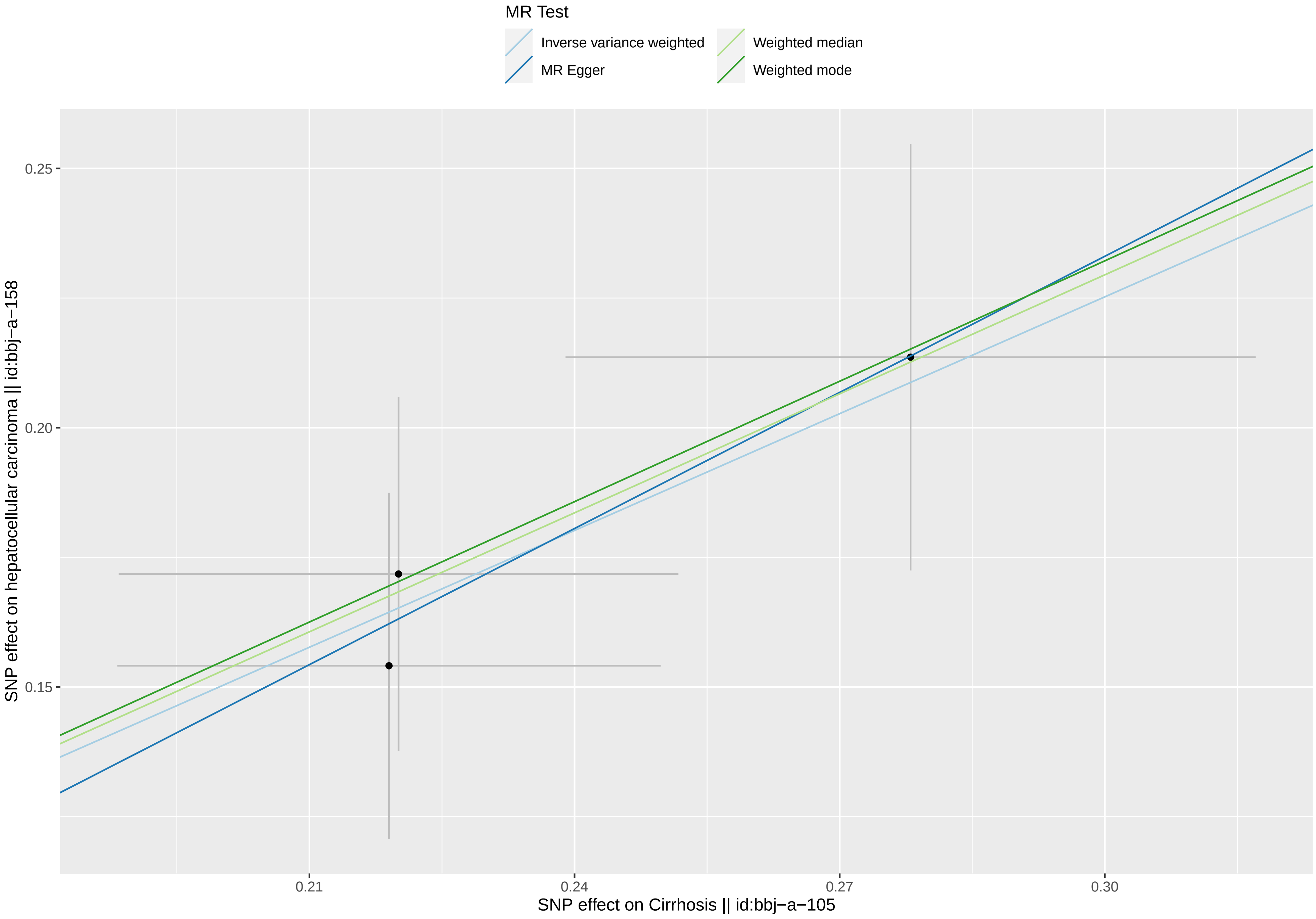

**Supplementary figure 10. Leave-one-out plot for the association between cirrhosis and hepatocellular carcinoma in East Asian populations**

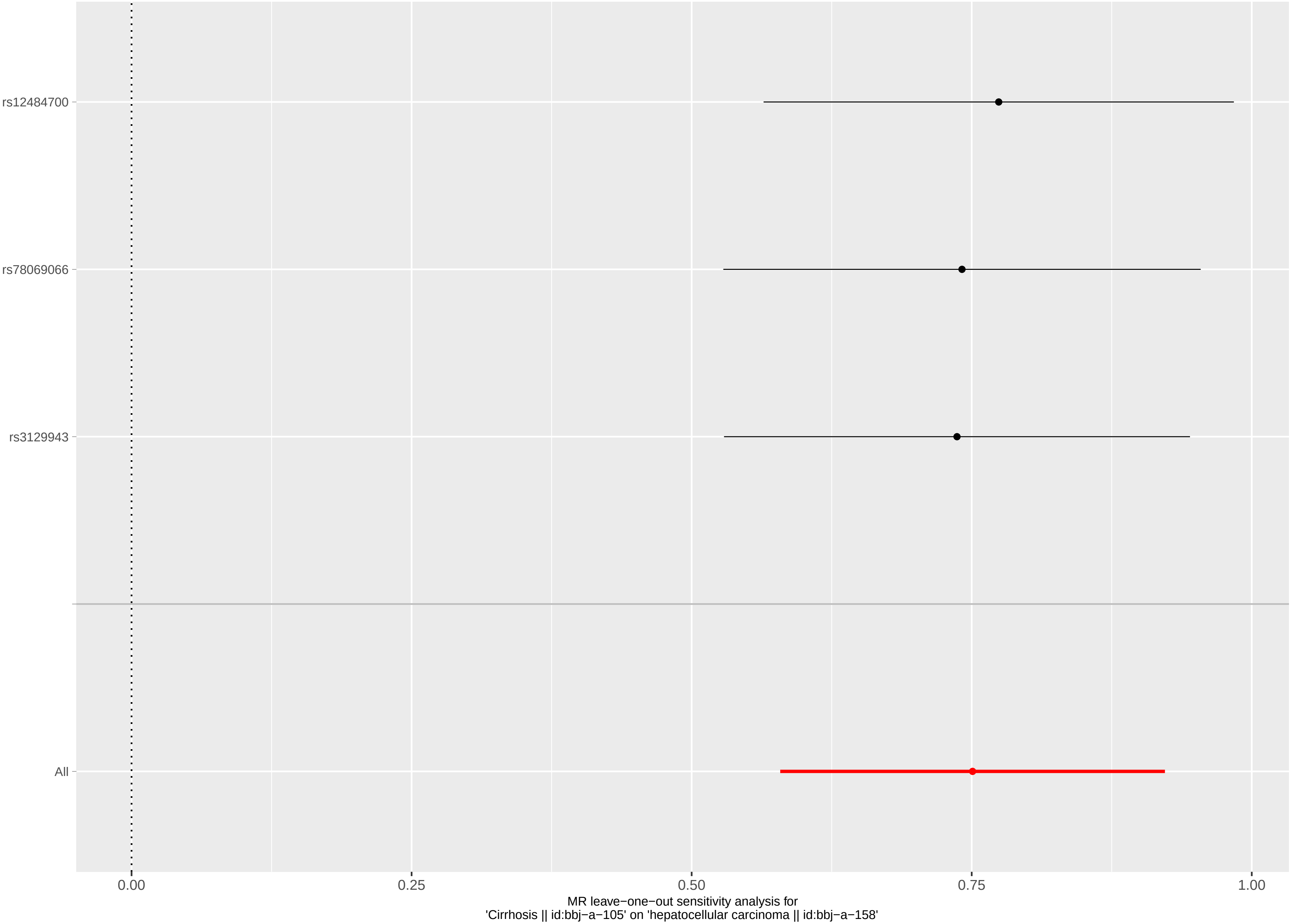

Supplement: Supplementary file 1 — Table S1. STROBE checklist of the current study. Table S2. MR estimates for the associations of NAFLD and cirrhosis with potential confounders in European and East Asian populations. Table S3. MR estimates for the associations of potential confounders with liver cancer in European and East Asian populations. Table S4. Characteristics of SNPs associated with NAFLD in European populations (after excluding overlapped sample). Table S5. MR estimates for the association of NAFLD with liver and intrahepatic bile ducts cancer in European populations (after excluding overlapped sample). Table S6. MR estimates for the association between cirrhosis and hepatocellular carcinoma in East Asian populations using individual SNPs. Figure S1. Scatter plot for the association between NAFLD and liver and intrahepatic bile ducts cancer in European populations. This plot showed the effect size of NAFLD on liver and intrahepatic bile ducts cancer using four MR univariable methods. The X‐axis is the effect of SNP on NAFLD (beta ± 95% confidence interval). The Y‐axis is the effect of SNP on liver and intrahepatic bile ducts cancer (beta ± 95% confidence interval). The line slope is the effect size. Figure S2. Leave‐one‐out plot for the association between NAFLD and liver and intrahepatic bile ducts cancer in European populations. This plot showed the effect size of NAFLD on liver and intrahepatic bile ducts cancer when excluding one SNP at a time from all used SNPs. The X‐axis is the effect size (beta ± 95% confidence interval). The Y‐axis is the ID of SNP. Figure S3. Scatter plot for the association between cALT and liver and intrahepatic bile ducts cancer in European populations. This plot showed the effect size of cALT on liver and intrahepatic bile ducts cancer using four MR univariable methods. The X‐axis is the effect of SNP on cALT (beta ± 95% confidence interval). The Y‐axis is the effect of SNP on liver and intrahepatic bile ducts cancer (beta ± 95% confidence interval). The line [file CNR2-7-e1913-s001.pdf]
